# Immunonutritional biomarkers in osteoarthritis: mechanistic insights and prognostic potential

**DOI:** 10.3389/fimmu.2026.1769089

**Published:** 2026-03-27

**Authors:** Jing Dan, Hua Ding, Lengtao Li, Qiang Wu

**Affiliations:** 1 Department of Sports Medicine and Rehabilitation, Affiliated Sports Hospital of Chengdu Sport University, Chengdu, China; 2 Department of Orthopedics, Hospital of Chengdu University of Traditional Chinese Medicine, Chengdu, China; 3 Department of Radiation Oncology, Cancer Center, West China Hospital, Sichuan University, Chengdu, China; 4 Lung Cancer Center and Institute, West China Hospital, Sichuan University, Chengdu, China

**Keywords:** immune regulation, immunometabolism, nutritional biomarkers, osteoarthritis, translational validation

## Abstract

Osteoarthritis (OA) is the most prevalent degenerative joint disease worldwide. Beyond structural injury to cartilage, synovium, and subchondral bone, OA is increasingly recognized as an immunometabolic disorder characterized by low-grade inflammation and dysregulated innate and adaptive immune responses. Nutritional biomarkers-derived from macro- and micronutrient status and metabolite signatures-can shape the OA inflammatory microenvironment by modulating macrophage polarization, T-cell subset balance, cytokine networks, and key signaling programs (e.g., NF-kappaB, JAK/STAT, NLRP3 inflammasome, and oxidative stress pathways), thereby influencing tissue catabolism and pain sensitization. This review reorganizes the literature around how clinically measurable nutritional biomarkers map onto immune-cell programs and core OA pathological processes, and critically appraises evidence strength and translational readiness. Most biomarker–OA links are supported primarily by mechanistic rationale and observational associations, while longitudinal and interventional validation remains limited and heterogeneous; key gaps include standardized assays and cutoffs, phenotype- and joint site–stratified prospective cohorts, and externally validated models demonstrating incremental prognostic utility beyond established clinical and imaging predictors.

## Graphical Abstract

**Figure:**
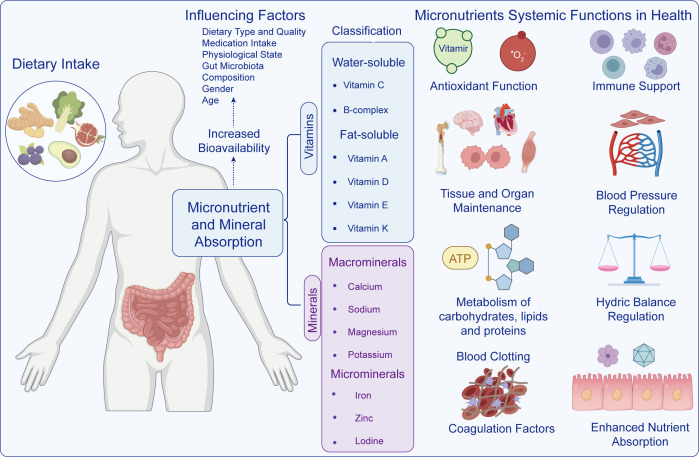
Nutritional biomarkers in osteoarthritis (OA): framework, mechanisms, evidence, and translation. This graphical abstract outlines the research rationale and current gaps (predominantly observational associations and limited standardized validation), classifies OA-related nutritional biomarkers into macronutrient- and micronutrient-related categories, and summarizes their potential immune-regulatory actions on innate and adaptive immunity (e.g., macrophage polarization, neutrophil activation, Th17/Treg balance, and B-cell function) via key inflammatory pathways including NF-κB and JAK/STAT, thereby contributing to synovitis and cartilage catabolism. It also summarizes representative human evidence (e.g., vitamin D, omega-3 fatty acids, and glycometabolism/AGEs), proposes a translational roadmap toward combined prognostic models, precision nutritional interventions, and optimized detection technologies, and highlights major challenges and prospects such as limited study designs, low biomarker specificity, inconsistent detection methods, and the need for phenotype-/joint site–stratified prospective studies, biomarker-enriched trials, and causal/clinical utility validation.

## 1 Introduction

Osteoarthritis is a group of chronic diseases characterized by degenerative changes of articular cartilage, chronic synovitis, subchondral bone remodeling, and joint dysfunction. Its incidence increases significantly with age, making it the leading cause of motor dysfunction and poor quality of life in middle-aged and elderly populations. Accumulating evidence indicates that immune dysregulation contributes to symptoms and, in some cohorts, to structural progression-particularly in inflammatory and early, synovitis-dominant phenotypes-yet OA is mechanistically heterogeneous and no single pathway universally dominates across patients, joints, or disease stages. In this context, activation of innate immune cells (e.g., macrophages and neutrophils), imbalance of adaptive immune subsets (T cells and B cells) (1, 2), and dysregulated cytokine networks can sustain a local inflammatory milieu that amplifies catabolic signaling, sensitizes nociceptors, and interacts with biomechanical and metabolic stressors. Consistent with this heterogeneity, OA has been conceptualized as a spectrum of partially overlapping phenotypes, including inflammation-dominant (synovitis/effusion, heightened cytokine and innate-immune activity) and metabolic/biomechanical phenotypes linked to obesity, insulin resistance, dyslipidemia, and altered adipokine signaling. Importantly, immune activation may be most evident in early or inflammatory presentations, whereas in other patients structural progression may be more strongly driven by mechanical overload, age-related matrix senescence, and metabolic stress, with inflammation acting as an amplifier rather than a universal primary cause. We therefore interpret immune dysregulation as phenotype- and stage-dependent and explicitly highlight areas of conflicting evidence throughout this review.

Nutrition is a key factor affecting body metabolism and immune function. The level of nutritional intake and metabolic status can directly influence the integrity of immune function. Nutritional biomarkers can be regarded as objective indicators to measure the body’s nutritional status. They mainly include metabolites of macronutrients, micronutrients and their derivatives, and metabolic intermediates, which can directly or indirectly reflect the relationship between nutrition, body immunity, and tissue damage. A growing number of studies have shown that nutritional biomarkers can not only measure the nutritional level of OA patients but also directly or indirectly regulate the immune cell functions of OA patients, affect the intensity of inflammatory responses (3, 4), and participate as a key link in the pathological process of OA. In this review, “nutritional biomarkers” refer to objectively measurable indicators in biospecimens (e.g., serum 25(OH)D, albumin, fatty-acid signatures, and SCFA profiles). In contrast, dietary intake patterns represent exposures, and supplementation represents an intervention; both may modify biomarker concentrations but should not be conflated with the biomarkers themselves. Accordingly, we discuss diet and supplementation only insofar as they inform biomarker sources/biological plausibility and provide interventional contexts in which biomarkers can stratify patients or monitor response. Conceptually, systemic nutritional biomarkers (typically assessed in blood or urine) integrate whole-body intake, absorption, storage, and inflammatory status and may not directly mirror the intra-articular milieu, whereas locally acting metabolites measured in synovial fluid, cartilage, or synovium can exert proximal effects on resident immune and stromal cells. Where available, we discuss both compartments and interpret concordance or discordance in light of physiology and sampling context.

Methodologically, nutritional status can be quantified at non-equivalent levels-dietary intake, circulating biomarkers, and joint/tissue concentrations-each with distinct sources of error. Dietary assessments (e.g., food-frequency questionnaires and recalls) are vulnerable to recall bias, misreporting, and limited capture of supplement use and food processing, while serum/plasma levels reflect bioavailability, redistribution, and acute-phase responses (e.g., inflammation-driven changes in albumin or lipid fractions) and can vary with fasting state, diurnal rhythms, assay platforms, and batch effects. Tissue- or synovial-fluid measurements may be more proximal to joint biology but are less feasible, are influenced by sampling timing and dilution, and are rarely available longitudinally, complicating causal inference and mediation testing.

Interpretation is further complicated by confounding and effect modification from genetic architecture, metabolic syndrome, medications, and the gut microbiome. Common genetic variants can influence baseline nutrient metabolism and circulating biomarker levels (e.g., vitamin D handling, fatty-acid desaturation, and lipid transport), and may also relate to OA susceptibility or pain phenotypes, creating gene-environment correlation. Metabolic syndrome and adiposity alter systemic inflammation and adipokine profiles, potentially obscuring biomarker specificity for joint processes. In parallel, microbiome composition and function shape short-chain fatty acids, bile-acid pools, endotoxemia, and immune tone; diet-induced microbiome shifts may therefore confound or mediate biomarker-OA associations. Accordingly, robust studies should harmonize phenotype definitions, measure key comorbidities, and apply appropriate adjustment/stratification (and, where possible, genetic or microbiome-informed analyses) when evaluating nutritional biomarkers in OA.

Despite growing interest in nutrition–OA links, several core gaps impede mechanistic understanding and clinical translation. First, existing studies are fragmented across nutrients, biospecimens, and endpoints, often reporting cross-sectional associations without harmonized definitions of OA phenotypes or inflammatory status. Second, most analyses do not explicitly map systemic nutritional biomarkers to joint-resident immune-cell programs (e.g., macrophage polarization, Th17/Treg balance, dendritic-cell maturation, and inflammasome activation), leaving the biomarker–pathology chain insufficiently articulated. Third, substantial methodological heterogeneity (sampling conditions, assay platforms, and cutoffs) and limited multivariable modeling/external validation hinder reproducible risk prediction, disease monitoring, and treatment-response stratification. Finally, interventional and longitudinal datasets that can test mediation and causality remain sparse, underscoring the need for a framework that integrates mechanistic plausibility with a practical validation roadmap.

However, systematic studies integrating nutritional biomarkers with OA-relevant immune circuits remain limited, and the clinical validation of these biomarkers for risk prediction, disease monitoring, or treatment-response stratification is still evolving. Therefore, clarifying how nutritional biomarkers reshape immune cell programs and inflammatory signaling in OA is essential to move from associative observations to mechanistically informed, testable clinical hypotheses. Accordingly, this review synthesizes OA-related nutritional biomarkers through an immunomodulatory framework (immune-cell targets and pathways), provides a critical appraisal of human evidence, and outlines practical recommendations for prospective validation and translation (3, 5). Specifically, we highlight where evidence is lacking, propose standardized outcome- and phenotype-aware validation strategies, and prioritize biomarkers most likely to provide actionable clinical utility.

Importantly, the current evidence linking nutritional biomarkers to OA is heterogeneous and is dominated by observational designs. Reported associations are frequently influenced by residual confounding (e.g., age, adiposity, metabolic comorbidities, medications, and physical activity) and potential reverse causality (reduced mobility and pain affecting diet and biomarker levels). Therefore, beyond summarizing mechanistic hypotheses, this review provides a critical appraisal of the strength and limitations of available evidence, highlights major sources of bias and measurement variability, and proposes priorities for phenotype-aware prospective validation and clinical translation.

## 2 Classification and characteristics of OA-related nutritional biomarkers

**Figure 1:**
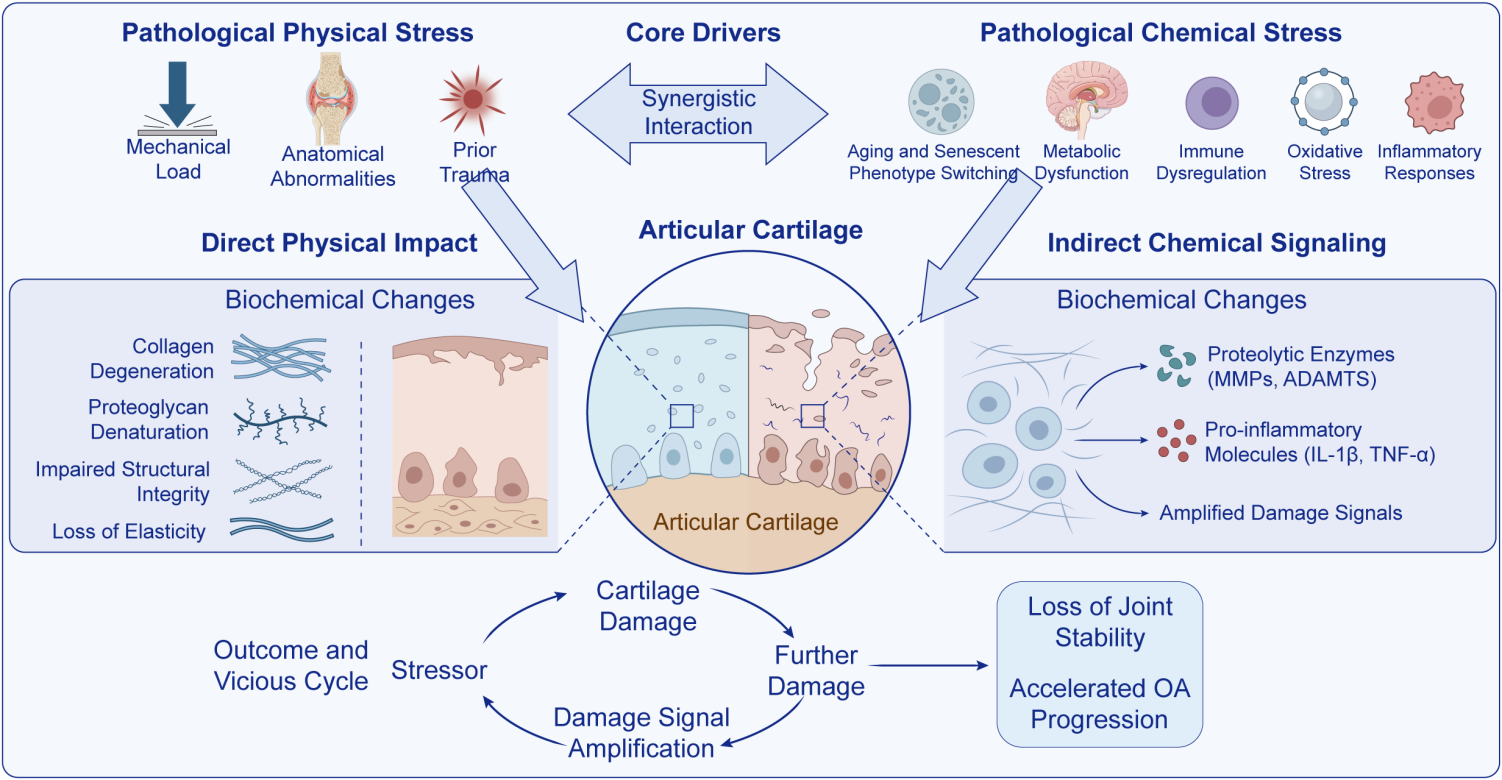
Integrated model of OA cartilage degeneration. Pathological physical stressors (excess mechanical load, anatomical abnormalities, and prior trauma) exert direct physical impact on articular cartilage, leading to collagen degeneration, proteoglycan denaturation, impaired structural integrity, and reduced elasticity. In parallel, pathological chemical stressors (aging/senescence-associated phenotype switching, metabolic dysfunction, immune dysregulation, oxidative stress, and inflammatory responses) promote indirect chemical signaling that induces proteolytic enzymes (MMPs, ADAMTS) and pro-inflammatory mediators (IL-1β, TNF-α), thereby amplifying damage signals. These synergistic drivers converge to disrupt cartilage homeostasis and establish a feed-forward cycle of cartilage damage, signal amplification, loss of joint stability, and accelerated OA progression. OA, osteoarthritis; MMPs, matrix metalloproteinases; ADAMTS, a disintegrin and metalloproteinase with thrombospondin motifs; IL-1β, interleukin-1 beta; TNF-α, tumor necrosis factor alpha.

Nutritional biomarkers are biological substances that reflect the intake, digestion, absorption, metabolism, and utilization of nutrients in the body, such as carbohydrates, lipids, proteins, vitamins, minerals, and mineral metabolites. Nutritional biomarkers of various nutrients form complex network interaction patterns with the inflammatory and immune mechanisms of OA. The flowchart of relevant factors involving nutritional biomarkers in OA is shown in **Figure 1**. To strengthen mechanistic continuity and reduce parallel listing across sections, we subsequently map these biomarkers onto core OA pathological processes (synovitis, cartilage catabolism, subchondral bone remodeling, and pain sensitization) by specifying the immune-cell subsets and signaling nodes they preferentially reprogram (**Table 1**). Notably, recent nutritional-epidemiology guidance emphasizes that candidate dietary biomarkers should be prioritized using explicit validation criteria (e.g., biological plausibility, dose–response, analytical robustness, stability, and reproducibility across populations); adopting this framework can reduce false-positive claims and strengthen translational prioritization of OA-related nutritional biomarkers (6). For clarity, throughout this review we distinguish biomarkers (measured indicators) from dietary exposures (intake patterns) and supplementation interventions that may influence biomarker levels.

**Table 1:** Immune-cell-centered mapping of representative nutritional biomarkers to key signaling hubs and OA pathological processes.

Representative nutritional biomarkers	Primary immune targets	Key signaling nodes	Dominant OA pathological process(es)
Omega-3 PUFAs (EPA/DHA) and pro-resolving mediators	Macrophage M2 polarization; reduced neutrophil recruitment; T-cell skewing toward Treg	PPARγ; reduced NF-κB/MAPK activation; specialized pro-resolving mediator signaling	Synovitis resolution; reduced cartilage catabolism
Omega-6 PUFA/arachidonic acid–derived eicosanoids (e.g., PGE2, leukotrienes)	Pro-inflammatory macrophage programming; neutrophil chemotaxis; nociceptor sensitization	COX/LOX pathways; EP receptors; NF-κB amplification loops	Synovitis amplification; pain sensitization
Vitamin D status (25[OH]D; 1,25[OH]2D3)	Macrophage inflammatory restraint; enhanced Treg function; restrained Th17 responses; DC tolerogenicity	VDR signaling; inhibition of NF-κB; modulation of JAK/STAT-mediated cytokine responses	Chronic synovitis control; cartilage protection; subchondral bone remodeling
Short-chain fatty acids (acetate/propionate/butyrate)	Treg induction; reduced inflammatory macrophage metabolism; gut–joint immune crosstalk	GPR41/43; HDAC inhibition; metabolic rewiring of innate immune cells	Systemic-to-local immune set-point; synovitis modulation; pain/inflammation coupling
Protein/amino-acid biomarkers (albumin, BCAAs, glutamine, arginine)	Immune-cell bioenergetics and proliferation; macrophage and T-cell functional fitness	mTOR–AMPK balance; NO synthesis (arginine–NOS); redox control (glutamine–GSH)	Inflammatory tone and repair capacity; disease susceptibility and progression
Micronutrient antioxidants (vitamin C/E; selenium-linked redox enzymes)	Reduced neutrophil- and macrophage-derived oxidative stress; preserved antigen-presenting-cell viability	ROS control; redox-sensitive NF-κB/MAPK signaling; antioxidant programs; GPX4-GSH (SLC7A11/xCT) lipid peroxidation checkpoint (ferroptosis control)	Cartilage oxidative injury; synovitis persistence; symptom severity; ferroptosis-linked ECM catabolism
Zinc and other trace elements	Phagocyte function and cytokine secretion; T- and B-cell maturation (dose-dependent)	Trace-element–dependent signaling and cytokine pathways	Immune competence *vs*. inflammatory amplification; synovitis and tissue damage
Advanced glycation end products (AGEs) and hyperglycemia-related markers	Pro-inflammatory macrophage activation; enhanced cytokine release; impaired resolution	RAGE signaling; NF-κB activation; oxidative-stress amplification	Cartilage catabolism; synovitis amplification; pain/inflammation coupling
Adipokines (leptin, adiponectin) and urate-related metabolites	Leptin-driven inflammatory T-cell/macrophage programs; adiponectin-linked counter-regulation; urate-triggered innate activation	JAK/STAT and NF-κB (adipokines); NLRP3 inflammasome (urate)	Metabolic–immune crosstalk; synovitis and flare-like inflammation; pain

### 2.1 Macronutrient-related biomarkers

Metabolites of macronutrients such as proteins, lipids, and carbohydrates in the body are important components of OA-related nutritional biomarkers. Protein metabolism biomarkers mainly include serum albumin, prealbumin, and branched-chain amino acids (leucine, isoleucine, valine) (7, 8). Serum albumin and prealbumin are classic biomarkers reflecting the body’s protein nutritional status and also general inflammatory biomarkers. Inflammation in OA patients can induce increased synthesis of acute-phase reactive proteins by the liver, leading to decreased synthesis of serum albumin and prealbumin. Hypoalbuminemia can further strengthen immune dysfunction by inhibiting the energy supply of immune cells. Branched-chain amino acids are important amino acid raw materials in muscle protein synthesis. Disorders of branched-chain amino acid metabolism can lead to insufficient proliferation and differentiation capacity of immune cells, interfere with the release of anti-inflammatory factors by immune cells, and exacerbate joint inflammation.

Lipid metabolism-related biomarkers mainly include unsaturated lipids (such as Omega-3 lipids, Omega-6 lipids), cholesterol, and triglycerides. Metabolites of Omega-3 lipids (such as eicosapentaenoic acid, docosahexaenoic acid) have clear anti-inflammatory effects, while metabolites of Omega-6 lipids (such as arachidonic acid) exacerbate inflammatory responses by promoting the synthesis of inflammatory mediators such as prostaglandins and leukotrienes (9, 10). The imbalance between the two is considered one of the important factors for the further amplification of inflammation in OA. As an important component of the cytoplasmic membrane, abnormal lipid metabolism will affect the stability and signal transduction of immune cell membranes, thereby regulating the activation of immune cells and the secretion of cytokines.

Carbohydrate metabolism-related biomarkers mainly include carbohydrate metabolites, blood glucose, glycated hemoglobin, insulin, and short-chain fatty acids (metabolites of dietary fiber). Hyperglycemia and hyperinsulinemia can induce oxidative stress responses, increase the level of advanced glycation end products (11, 12), activate inflammation-related signaling pathways, aggravate articular cartilage damage, and exacerbate immune-inflammatory responses. Short-chain fatty acids are substances produced by dietary fiber through the action of intestinal flora, which reach joint tissues through the bloodstream and affect immune cell activity. Changes in their metabolites are closely related to the severity of OA.

### 2.2 Micronutrient-related biomarkers

**Figure 2:**
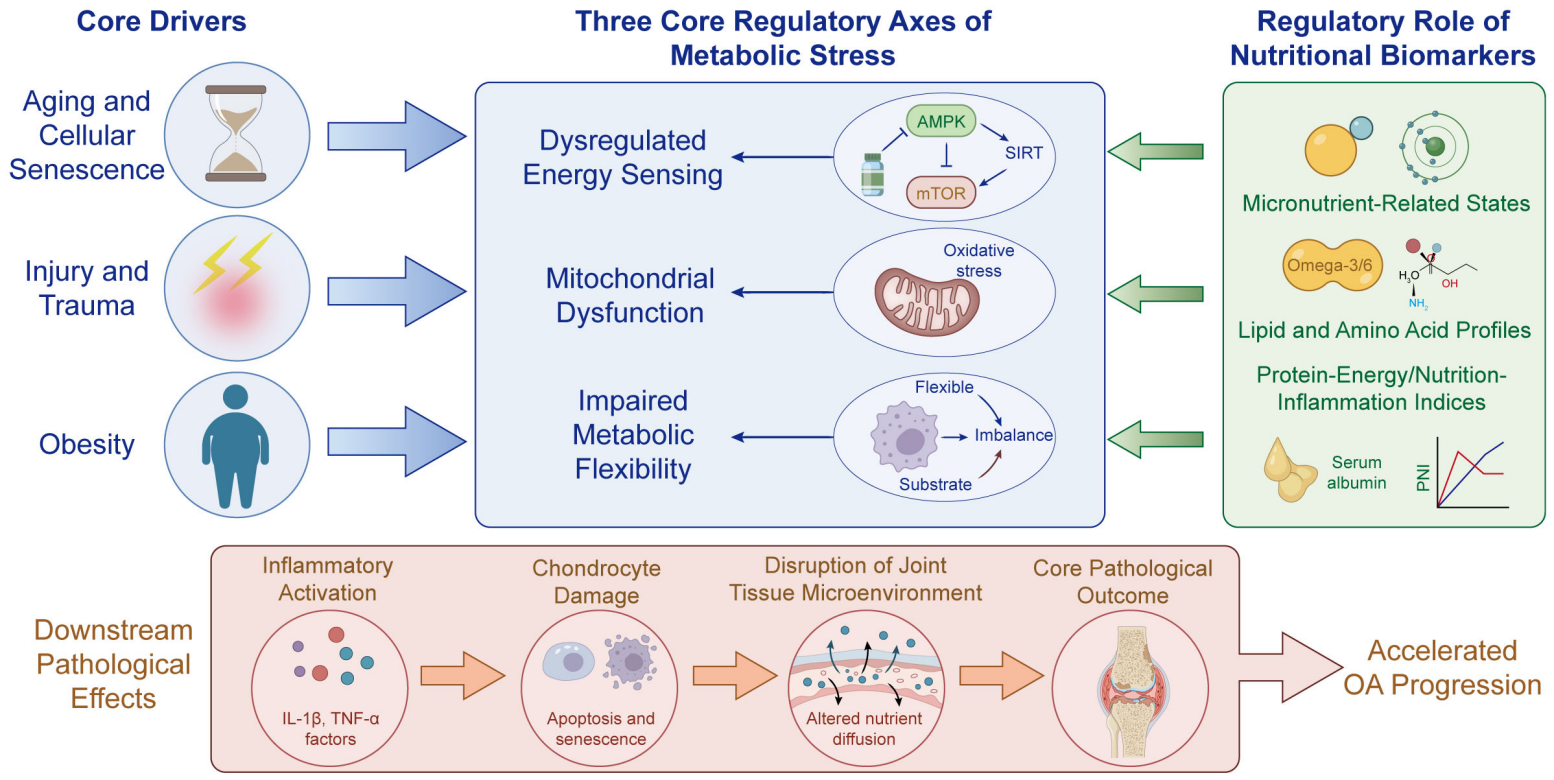
Overview of micronutrient intake, absorption, and systemic functions relevant to joint health. Dietary micronutrients (vitamins and minerals) are absorbed in the gastrointestinal tract, and their bioavailability is shaped by diet quality, medications, physiological state, gut microbiota, sex, and age. Micronutrients are grouped into water-soluble and fat-soluble vitamins (A, D, E, K) and macro-/trace minerals. After uptake, they support antioxidant defense, immunoregulation, cellular differentiation and tissue maintenance, energy and macronutrient metabolism, coagulation, fluid balance, and blood pressure regulation. Collectively, these systemic actions provide a mechanistic basis for how micronutrient status may influence OA susceptibility and progression.

Micronutrients include vitamins and minerals, whose role in regulating immune function cannot be ignored. Biomarkers formed by their metabolic status are also related to the occurrence and development of OA. The role of micronutrient-related biomarkers in healthy individuals is shown in **Figure 2**. Vitamin-related biomarkers include vitamin D, vitamin C, vitamin E, and B vitamins. Vitamin D is a vitamin with hormonal activity. Its active form (1,25-dihydroxyvitamin D3) can bind to vitamin D receptors on immune cells, regulate the functions of macrophages, T cells, and B cells, inhibit the release of pro-inflammatory factors (13, 14), promote the production of anti-inflammatory factors, regulate the metabolism of chondrocytes, and reduce cartilage damage. Vitamin C is a strong antioxidant that can scavenge reactive oxygen species in the joint, reduce oxidative stress damage, promote collagen synthesis, and maintain the structural stability of cartilage. A decrease in serum levels is associated with an increased risk of OA. Vitamin E has antioxidant and anti-inflammatory effects, which can protect immune cells and joint tissue cells from damage by inhibiting lipid peroxidation. B vitamins (B6, B12, folic acid) are involved in the synthesis of energy substances and nucleic acids in the body (15, 16). Their deficiency can lead to decreased proliferation capacity of immune cells and imbalanced regulation of inflammatory responses, thereby exacerbating the occurrence of OA.

Mineral biomarkers include calcium, phosphorus, zinc, selenium, copper, etc. Calcium and phosphorus are involved in the composition of bone and cartilage, and the balance of calcium-phosphorus metabolism influences the structural integrity of articular cartilage and subchondral bone. Imbalance of calcium and phosphorus metabolism may contribute to subchondral bone sclerosis and osteophyte formation, thereby accelerating OA progression. Zinc is a structural component and cofactor of multiple enzymes and is closely related to immune-cell development and activation (17, 18). Zinc deficiency can reduce macrophage phagocytic capacity, impair T-cell proliferation, and disturb inflammatory mediator secretion, potentially aggravating joint inflammation. Selenium is an essential component of glutathione peroxidase with antioxidant activity and has been reported to associate with OA symptom severity and functional outcomes in observational studies, although causal and prognostic interpretations require longitudinal validation. Copper participates in oxidase synthesis and may influence tissue repair and regeneration through regulation of oxidative stress and inflammatory responses.

### 2.3 Metabolite-derived nutritional biomarkers

**Figure 3:**
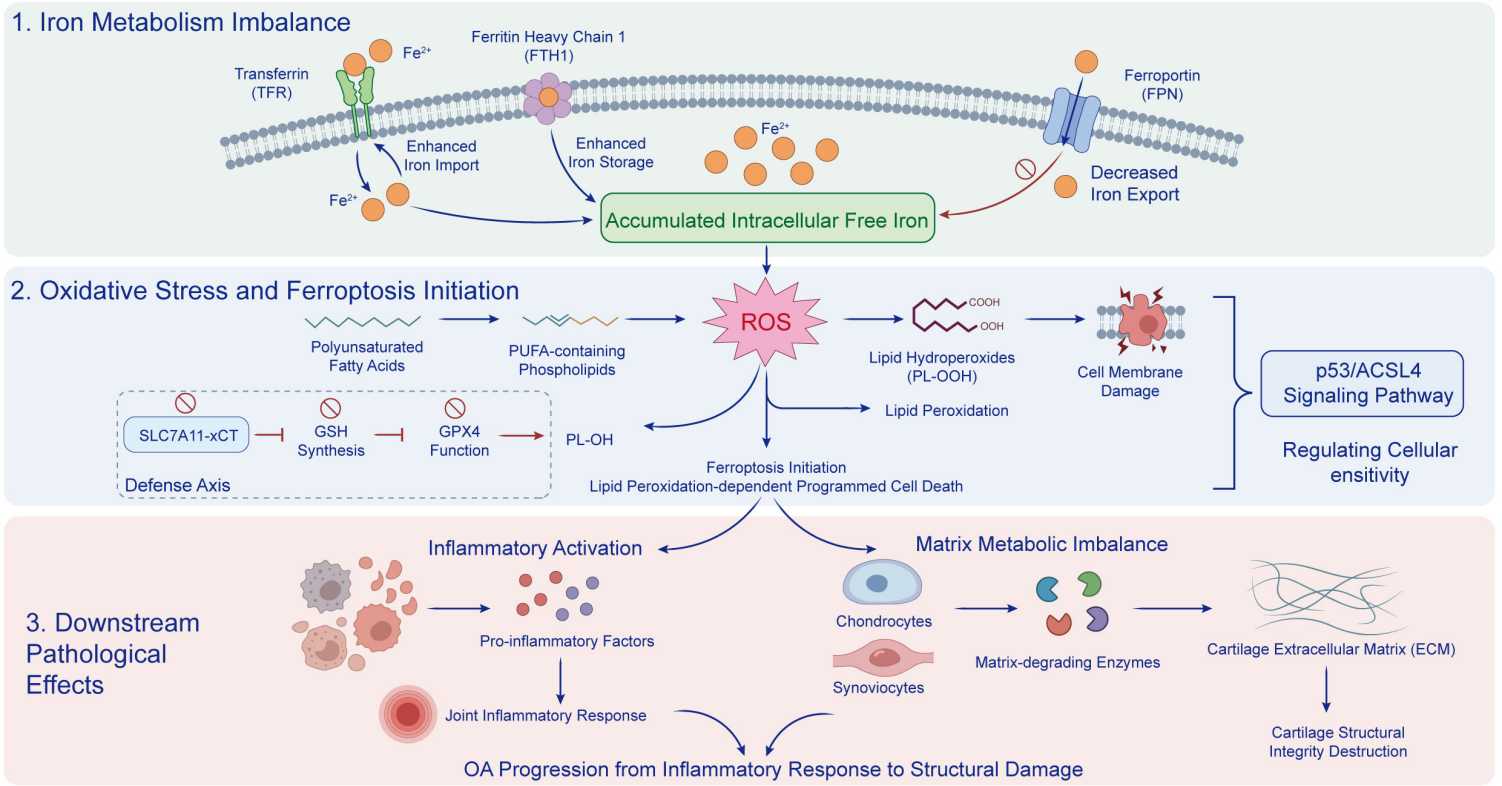
Metabolic-stress circuitry linking nutritional biomarkers to OA pathogenesis. This schematic illustrates how major OA-associated drivers, including aging and cellular senescence, injury and trauma, and obesity, converge on three core regulatory axes of metabolic stress: dysregulated energy sensing, mitochondrial dysfunction, and impaired metabolic flexibility. Dysregulated energy sensing is represented by the AMPK–SIRT–mTOR network, while mitochondrial dysfunction is coupled to oxidative stress, and impaired metabolic flexibility is characterized by substrate imbalance and reduced adaptive capacity. Nutritional biomarkers are positioned as upstream modulators of these axes, encompassing micronutrient-related states, lipid and amino acid profiles, and protein-energy/nutrition-inflammation indices such as serum albumin and PNI. Perturbation of these interconnected pathways promotes inflammatory activation, chondrocyte apoptosis and senescence, and disruption of the joint tissue microenvironment, ultimately contributing to the core pathological outcome of accelerated OA progression. OA, osteoarthritis; AMPK, AMP-activated protein kinase; SIRT, sirtuin; mTOR, mechanistic target of rapamycin; IL-1β, interleukin-1 beta; TNF-α, tumor necrosis factor alpha; PNI, prognostic nutritional index.

In addition to the above nutritional metabolism biomarkers directly related to nutrients, some derivatives produced by nutritional metabolism can also serve as biomarkers for the correlation between nutritional status and OA, mainly including amino acid metabolism derivatives, lipid metabolism derivatives, and carbohydrate metabolism derivatives. Among them, amino acid metabolism derivatives such as glutamine and arginine. Glutamine is the main energy substance for immune cells. A decrease in glutamine content can cause immune cell dysfunction and increase the occurrence of inflammatory responses. Arginine can regulate immune cell activation and vasodilation through the synthesis of nitric oxide (19, 20). Disorders of arginine metabolism are related to the aggravation of joint inflammation. The classification of metabolite-derived nutritional biomarkers and their effects on OA are shown in **Figure 3**.

Lipid metabolism derivatives include prostaglandins, leukotrienes, adiponectin, leptin, etc. Among them, prostaglandins and leukotrienes are important inflammatory mediators. Their formation is closely related to the metabolism of unsaturated fatty acids, which can stimulate vasodilation, increase vascular permeability, infiltrate immune cells, and accelerate joint synovitis lesions. Adiponectin and leptin, as adipokines secreted by adipose tissue, not only participate in regulating energy metabolism but also regulate immune cell functions. Adiponectin can exert anti-inflammatory effects (9, 21), while leptin can induce the secretion of pro-inflammatory factors. The imbalance between the two plays an important role in the immune imbalance of OA.

Metabolic end products such as advanced glycation end products and fructose end products. Advanced glycation end products bind to receptors on the cell surface to activate inflammatory signaling pathways, promote the release of pro-inflammatory factors, or directly damage chondrocyte functions and accelerate cartilage degradation. Fructose end products such as uric acid. Increased uric acid concentration leads to the deposition of urate crystals in joint tissues, activating the innate immune system and inducing acute inflammatory responses. Persistent hyperuricemia can accelerate the development of OA (22, 23).

## 3 Immune regulatory mechanisms of nutritional biomarkers in OA

Building on the classification above, we adopt an immune-cell–centered framework to link nutritional biomarkers to OA lesions. Rather than re-categorizing biomarkers and immune effects in parallel, we emphasize how representative biomarkers reprogram specific immune-cell subsets (e.g., macrophage polarization and inflammasome activation, neutrophil recruitment, dendritic-cell maturation and antigen presentation, and Th17/Treg balance) and converge on shared signaling hubs (NF-κB, MAPK, JAK/STAT, NLRP3, PPARγ, and VDR). These immune changes drive four interconnected pathological processes—synovitis, cartilage catabolism, subchondral bone remodeling, and pain sensitization—thereby providing a progressively organized mechanistic narrative. To avoid overgeneralization, we explicitly distinguish evidence derived from human OA cohorts/trials, preclinical animal models, and *in vitro* experiments. Where mechanistic statements are supported mainly by preclinical or cell-based data, we state this explicitly and interpret the findings as biological plausibility rather than clinical proof. We also contextualize exposure levels by differentiating nutritional-range status or supplementation from pharmacologic dosing that is sometimes required to elicit antioxidant or immunomodulatory effects in experimental systems.

### 3.1 Immune-cell-centered framework linking nutritional biomarkers to major OA pathological processes

OA is increasingly recognized as an immunometabolic disease in which nutritional status shapes the inflammatory set-point of the joint. Accordingly, nutritional biomarkers influence OA progression not as isolated factors but by tuning the behavior of synovial-resident and infiltrating immune cells and the signaling pathways they engage.

OA-specific immunopathology is dominated by synovial macrophage activation and innate immune sensing of cartilage-derived damage signals (e.g., fibronectin fragments, HMGB1, S100A8/A9), which engage TLR2/4 and RAGE to trigger IKK–NF-κB and MAPK cascades and, in some contexts, NLRP3 inflammasome activation with IL-1β maturation. These signals propagate across the synovitis–cartilage–subchondral bone axis: cytokines and lipid mediators promote chondrocyte catabolism (MMP13/ADAMTS5) and osteochondral remodeling via RANKL-driven osteoclastogenesis and osteoblast TGF-β/Wnt programs. Accordingly, we map each nutritional biomarker onto joint-relevant immune pathways and tissue readouts to reduce repetition and strengthen OA-specific mechanistic inference.

First, during synovitis initiation and amplification, cartilage- and matrix-derived damage-associated molecular patterns activate synovial macrophages and dendritic cells, which orchestrate cytokine production and leukocyte recruitment. Anti-inflammatory biomarkers (e.g., omega-3-derived mediators, vitamin D, and short-chain fatty acids) tend to restrain NF-κB/MAPK-driven inflammatory programs, promote resolution-oriented macrophage states, and reduce neutrophil influx, whereas pro-inflammatory metabolic cues (e.g., omega-6-derived eicosanoids and AGE–RAGE signaling) reinforce feed-forward inflammation.

Second, for cartilage catabolism, immune-derived cytokines (TNF-α, IL-1β, and IL-17) and reactive oxygen species activate catabolic signaling in chondrocytes, upregulating matrix-degrading enzymes (MMPs and ADAMTS) and promoting chondrocyte senescence or apoptosis. Nutritional biomarkers that enhance regulatory T-cell activity, limit Th17 polarization, and improve redox balance can indirectly dampen these catabolic cascades, linking immune modulation to structural cartilage preservation.

Third, in subchondral bone remodeling and osteophyte formation, immune–bone crosstalk (including cytokine-driven osteoclastogenesis and osteoblast activity) connects synovitis with biomechanical instability. Finally, inflammatory mediators and lipid-derived prostanoids contribute to peripheral and central sensitization, coupling synovitis to pain. The following sections therefore dissect biomarker effects at the level of immune-cell subsets, signaling hubs, and immune–tissue communication to explain their roles across these pathological processes.

### 3.2 Modulation of innate immune cells: macrophage polarization, neutrophil activation, and dendritic-cell maturation in synovitis

**Figure 4:**
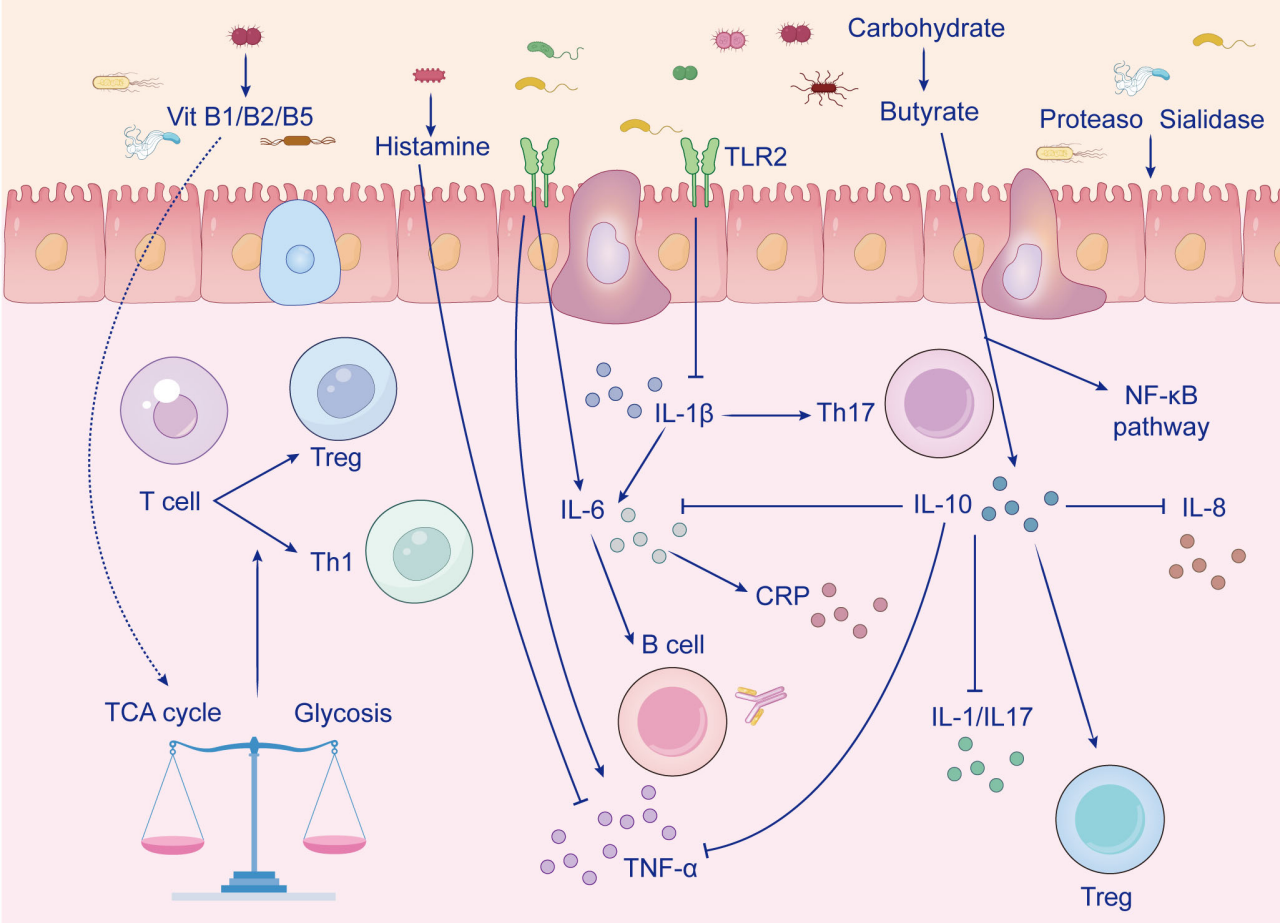
Iron overload-driven ferroptosis promotes inflammatory catabolism and extracellular matrix degradation in OA. This schematic illustrates how disruption of iron metabolism contributes to ferroptosis-associated OA progression. In the upper panel, enhanced iron import through transferrin receptor (TFR), increased iron storage involving ferritin heavy chain 1 (FTH1), and reduced iron export via ferroportin (FPN) collectively lead to intracellular free iron accumulation. In the middle panel, excess iron promotes reactive oxygen species (ROS) generation and lipid peroxidation of polyunsaturated fatty acid (PUFA)-containing phospholipids, resulting in lipid hydroperoxide (PL-OOH) accumulation, cell membrane damage, and initiation of ferroptotic cell death. This process is further facilitated by impairment of the antioxidant defense axis, including suppression of system Xc⁻/SLC7A11, glutathione (GSH) synthesis, and GPX4 function, while the p53/ACSL4 signaling pathway modulates cellular sensitivity to ferroptosis. In the lower panel, ferroptosis-associated oxidative injury drives inflammatory activation and matrix metabolic imbalance, characterized by increased pro-inflammatory mediators, enhanced joint inflammatory responses, and induction of matrix-degrading enzymes by chondrocytes and synoviocytes, ultimately leading to extracellular matrix breakdown, cartilage structural damage, and progressive OA deterioration. OA, osteoarthritis; TFR, transferrin receptor; FTH1, ferritin heavy chain 1; FPN, ferroportin; ROS, reactive oxygen species; PUFA, polyunsaturated fatty acid; PL-OOH, lipid hydroperoxide; SLC7A11, solute carrier family 7 member 11; GSH, glutathione; GPX4, glutathione peroxidase 4; ACSL4, acyl-CoA synthetase long-chain family member 4; ECM, extracellular matrix.

Innate immune activation is a primary driver of synovitis and an upstream amplifier of cartilage and subchondral bone injury in OA. Nutritional biomarkers described in Section 2 can reprogram innate immune cell metabolism and activation thresholds, thereby influencing whether synovial inflammation resolves or persists. The regulation of OA immune cells by nutritional biomarkers is shown in **Figure 4**. Below, we highlight macrophage polarization, neutrophil effector functions, and dendritic-cell maturation as key entry points through which nutritional cues shape OA pathology.

In OA synovitis, macrophage-derived IL-1β, TNF-α, and IL-6 are key upstream signals that couple synovial inflammation to cartilage catabolism and subchondral remodeling; these outputs are typically amplified by IKK–NF-κB/MAPK signaling and, in pro-oxidant contexts, by NLRP3 inflammasome priming. Therefore, pathway-specific biomarker effects are most informative when explicitly anchored to these nodes (e.g., NF-κB, p38/JNK, JAK/STAT3, NLRP3, and COX-2–PGE2).

Macrophages play a double-edged role in the pathological process of OA. M1 macrophages release a large number of pro-inflammatory factors (including tumor necrosis factor-α, interleukin-6, interleukin-1β), aggravating joint inflammation and cartilage damage. M2 macrophages can release anti-inflammatory factors (including interleukin-10, transforming growth factor-β) to promote tissue repair (24, 25). Nutritional biomarkers can influence macrophage programming: omega-3-related lipid signatures may favor resolution-oriented macrophage states and restrain pro-inflammatory cytokine production via PPARγ-linked pathways, while vitamin D status can modulate macrophage inflammatory tone through VDR-dependent checkpoints. Short-chain fatty acids have been shown predominantly in animal models and *in vitro* systems to rewire macrophage metabolism and cytokine secretion via G protein-coupled receptors and HDAC inhibition; however, whether nutritional-range variation produces comparable intra-articular effects in human OA remains to be demonstrated.

Pathway anchoring examples in OA: omega-3 lipid signatures can engage FFAR4/GPR120 and PPARγ and promote pro-resolving mediator signaling (e.g., ALX/FPR2 and ChemR23), thereby limiting IKK–NF-κB activity and reducing NLRP3-dependent IL-1β release, which in turn attenuates COX-2/PGE2 and matrix-degrading enzyme induction in the cartilage compartment. Vitamin D biomarkers (25[OH]D/1,25[OH]2D) act through VDR to increase inhibitory checkpoints (e.g., IκBα and SOCS1/3), dampening IL-6–JAK/STAT3 amplification and supporting tolerogenic DC programs that restrain Th17-skewing cues. SCFAs (butyrate/propionate) signal via GPR43/41 and HDAC inhibition to enhance IL-10/Foxp3 programs and rewire macrophage metabolism toward oxidative phosphorylation, shifting synovitis toward resolution. Conversely, AGE/hyperglycemia-related biomarkers can activate RAGE–NF-κB/p38 and ROS amplification, favoring inflammatory macrophage programs and inflammasome priming.

Neutrophils are the earliest immune cells to infiltrate inflammatory sites and can release proteases (such as matrix metalloproteinases) and reactive oxygen species, which may contribute to synovial inflammation and cartilage injury. With respect to antioxidant vitamins, vitamin C and vitamin E are plausible modulators of neutrophil-derived oxidative stress; however, direct evidence demonstrating that nutritional-range vitamin C/E status restores antioxidant defenses within human OA cartilage or synovium remains limited. Many cytoprotective antioxidant effects are derived from *in vitro* experiments using supra-physiologic concentrations, and the extent to which dietary supplementation achieves comparable intra-articular exposure is uncertain. Zinc can influence neutrophil activation and degranulation in a dose-dependent manner and may reduce excessive protease release, potentially mitigating tissue injury (25, 26). Omega-3 fatty acids can inhibit neutrophil chemotaxis and infiltration, thereby reducing the intensity of inflammatory responses.

Dendritic cells, as key cells connecting innate immunity and adaptive immunity, their maturation and antigen-presenting function have a key impact on downstream immune responses. Nutritional biomarkers can regulate the functions of dendritic cells: vitamin D inhibits the maturation of dendritic cells, reduces their antigen-presenting capacity, and inhibits the excessive activation of adaptive immune responses. Folic acid can promote the development and maturation of dendritic cells by participating in nucleic acid synthesis, enhancing their immune regulatory functions (27). Selenium can protect dendritic cells from oxidative stress damage through antioxidant effects, maintaining their antigen-presenting capacity. **Figure 4**.

Within OA, ferroptosis is most appropriately interpreted as an immunometabolic amplifier embedded in the joint microenvironment and sustained by inter-tissue crosstalk. Activated synovial macrophages and neutrophils increase labile iron and ROS and provide IL-1β/TNF-α cues that can lower the SLC7A11–GSH–GPX4 antioxidant checkpoint in chondrocytes and synoviocytes, thereby sensitizing them to lipid-peroxidation-driven death. In turn, ferroptotic joint cells may release oxidized phospholipids and danger signals that amplify TLR/RAGE–NF-κB programs in synovial myeloid cells, reinforcing synovitis and cartilage matrix catabolism (28, 29).

Several clinically measurable nutritional biomarkers map directly onto ferroptosis control points. Selenium status (or glutathione peroxidase activity) reflects the capacity of selenium-dependent GPX4 to detoxify phospholipid hydroperoxides, while vitamin E and other lipid-phase antioxidants raise the threshold for membrane lipid peroxidation. Biomarkers related to cyst(e)ine availability and systemic redox balance (e.g., glutathione-related indices, albumin/prealbumin as integrated nutritional-inflammatory surrogates) inform substrate supply for GSH synthesis and thus xCT/SLC7A11-GPX4 function. Finally, iron-related biomarkers (ferritin, transferrin saturation, hepcidin) and PUFA signatures (omega-6 *vs* omega-3 composition) can plausibly stratify ferroptotic susceptibility by modulating labile iron load and the pool of peroxidizable phospholipids in the joint.

### 3.3 Adaptive immune skewing: Th17/Treg balance and B-cell activation in chronic inflammation and cartilage catabolism

Adaptive immune dysregulation contributes to the transition from episodic synovitis to chronic, self-sustaining inflammation and can potentiate cartilage catabolism through sustained cytokine pressure. A nutritionally tuned shift in T-cell differentiation (particularly the Th17/Treg axis) and B-cell activation status provides a mechanistic bridge between systemic metabolic states and local joint immune balance.

In OA, adaptive immune signatures vary by phenotype, but Th17-associated cytokines and reduced regulatory checkpoints have been reported in synovial tissue and fluid in inflammatory subsets. Mechanistically, IL-23-JAK/STAT3-RORγt programs favor Th17 differentiation, whereas TGF-β/SMAD and epigenetic control (e.g., SCFA-driven HDAC inhibition) support Foxp3+ Treg stability; mapping nutritional biomarkers onto these specification nodes clarifies how systemic status could translate into joint cytokine pressure (IL-17A/IL-22) and downstream chondrocyte catabolism.

T cell subgroups can be divided into helper T cells (Th1, Th2, Th17), regulatory T cells (Treg), etc. Their imbalance is an important cause of chronic inflammation in OA. Pro-inflammatory factors secreted by Th1 and Th17 cells exacerbate joint inflammation in OA (30), while anti-inflammatory factors secreted by Th2 and Treg cells inhibit inflammation. Nutritional biomarkers can regulate the differentiation of T cell subgroups: Omega-3 fatty acids balance pro-inflammatory and anti-inflammatory immune responses by inhibiting the differentiation of Th1 and Th17 cells and promoting the growth of Th2 and Treg cells. Vitamin D inhibits the differentiation of Th17 cells, promotes the proliferation and function of Treg cells, and enhances immune suppression by activating the vitamin D receptor signaling pathway. Short-chain fatty acids affect systemic T cell subgroup imbalance by regulating intestinal mucosal immunity, promoting the differentiation of Treg cells, and inhibiting the activity of pro-inflammatory T cells (30).

B cells can secrete antibodies, cytokines, etc., to participate in immune-inflammatory responses. Their abnormal activation can promote the production of autoantibodies that attack joint tissues, exacerbating inflammatory damage. Nutritional biomarkers can regulate the activation functions of B cells: vitamin D inhibits the proliferation and differentiation of B cells, reducing the production of antibodies, including autoantibodies. Zinc promotes the development and maturation of B cells, enhancing immune function, but excessive zinc can lead to excessive activation of B cells and aggravate inflammatory damage (31). Folic acid deficiency results in low proliferation capacity of B cells and reduced antibody secretion, failing to fully exert the integrity of immune responses.

### 3.4 Cytokine and signaling hubs linking nutritional biomarkers to OA inflammation

**Figure 5:**
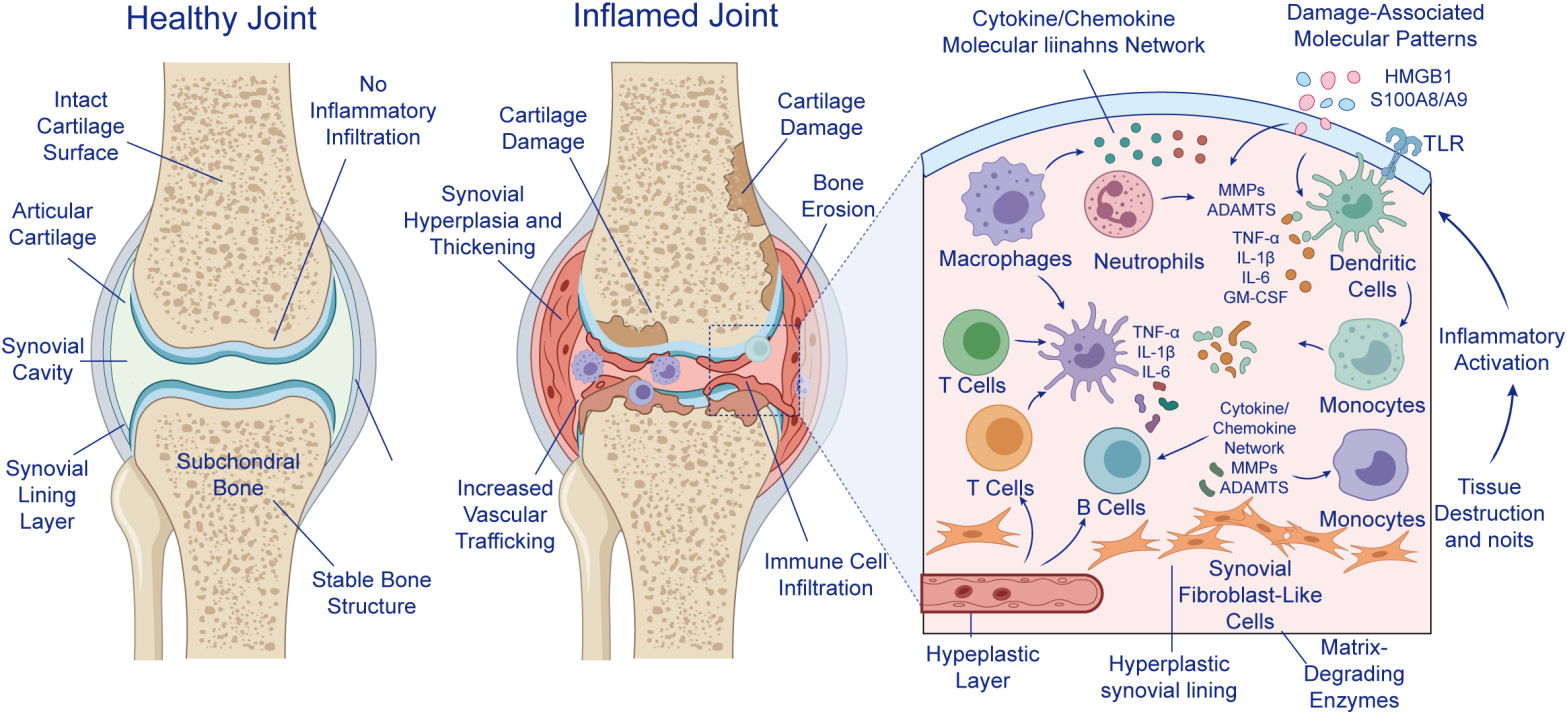
Gut microbiota–derived metabolites shape systemic immune tone relevant to OA.

At the molecular level, diverse nutritional biomarkers converge on a limited set of cytokine and stress-response signaling hubs that govern immune-cell effector programs and chondrocyte catabolic transcription. Key nodes include NF-κB and MAPK pathways, JAK/STAT-mediated cytokine amplification, NLRP3 inflammasome activation (with downstream IL-1β release), and resolution-associated programs such as PPARγ and VDR signaling. Framing cytokine regulation through these hubs helps explain why distinct biomarker classes can produce overlapping pathological outcomes. The cytokine balance regulated by nutritional biomarkers is shown in **Figure 5**.

These hubs also exhibit tissue-specific roles within the joint: NF-κB/MAPK activation in synovial macrophages and fibroblasts drives IL-1β/TNF-α/IL-6 that act on chondrocytes to induce catabolic gene programs (MMP13, ADAMTS5) and senescence, whereas IL-6 trans-signaling (sIL-6R/gp130) and COX-2–PGE2 can promote osteoclastogenic RANKL expression and sensitize nociceptors. Accordingly, biomarkers linked to these nodes can be interpreted as more reflective of synovitis-dominant, metabolic/AGE-driven, or osteochondral-remodeling phenotypes, rather than a single generic ‘anti-inflammatory’ effect.

Pro-inflammatory factors, such as TNF-α, IL-6, IL-1β, and IL-17, can stimulate chondrocytes to secrete MMPs, accelerating cartilage matrix degradation, and recruit immune cell infiltration to exacerbate joint inflammation. Nutritional biomarkers may modulate these pro-inflammatory signals: omega-3 fatty acid metabolites can reduce the production of pro-inflammatory prostaglandins and leukotrienes by competing with arachidonic acid–derived pathways (32). Vitamin C has been proposed to attenuate redox-sensitive activation of the nuclear factor-κB pathway based largely on *in vitro* and animal evidence, but OA-specific human data directly linking vitamin C status to reduced NF-κB activity, synovial cytokine burden, or cartilage catabolism are limited and inconsistent. Selenium-related biomarkers may reflect antioxidant selenoprotein capacity and have been associated with symptoms in some cohorts; nevertheless, mechanistic inference for cytokine suppression within human OA joints remains indirect and should be interpreted cautiously.

Anti-inflammatory factors such as interleukin-10, transforming growth factor-β, and interleukin-4 can inhibit the excessive activation of immune cells, promote the repair and regeneration of chondrocytes, and reduce joint inflammation. Nutritional biomarkers can promote the expression of these anti-inflammatory factors: Omega-3 fatty acids can promote macrophages and T cells to secrete interleukin-10 and transforming growth factor-β. Vitamin D can enhance the ability of Treg to secrete interleukin-10. Short-chain fatty acids can promote the expression of anti-inflammatory factors by stimulating immune cell signaling pathways (27, 28).

Nutritional biomarkers can also regulate the expression of cytokine receptors and signal transduction, affecting the biological effects of cytokines. For example, vitamin D can regulate the expression of pro-inflammatory factor receptors on the surface of immune cells, reducing the sensitivity of cells to pro-inflammatory factors. Zinc can participate in the regulation of cytokine signaling pathways, strengthen the signal transduction of anti-inflammatory factors, and inhibit the role of pro-inflammatory factors (33, 34).

This schematic illustrates how gut microbiota–derived metabolites and microbial enzymatic products influence host immune regulation in ways relevant to OA pathogenesis. Beneficial microbial metabolites, including butyrate generated from carbohydrate fermentation and vitamins such as B1, B2, and B5, are shown to support epithelial homeostasis, modulate cellular metabolism, and promote immune balance by influencing the TCA cycle–glycolysis axis and T-cell differentiation. These effects favor regulatory immune phenotypes, including Treg responses, while constraining excessive pro-inflammatory activation. In parallel, microbial signals such as histamine and Toll-like receptor 2 (TLR2)-related stimulation influence epithelial and immune cell crosstalk, thereby shaping downstream cytokine networks involving IL-1β, IL-6, IL-8, IL-10, IL-17, TNF-α, and C-reactive protein (CRP), as well as B-cell and Th1/Th17-associated responses. Conversely, dysbiosis-associated products, including proteases and sialidases, may impair barrier integrity and activate NF-κB-linked inflammatory signaling. Collectively, the figure highlights a mechanistic link between gut microbial metabolism, mucosal immune regulation, and systemic inflammatory pathways that may contribute to OA initiation and progression.

Abbreviations: OA, osteoarthritis; TLR2, Toll-like receptor 2; NF-κB, nuclear factor kappa B; Treg, regulatory T cell; Th1, T helper 1 cell; Th17, T helper 17 cell; IL, interleukin; TNF-α, tumor necrosis factor alpha; CRP, C-reactive protein; TCA cycle, tricarboxylic acid cycle.

### 3.5 Immune-tissue cell crosstalk driving cartilage damage and subchondral remodeling (with implications for pain)

**Figure 6:**
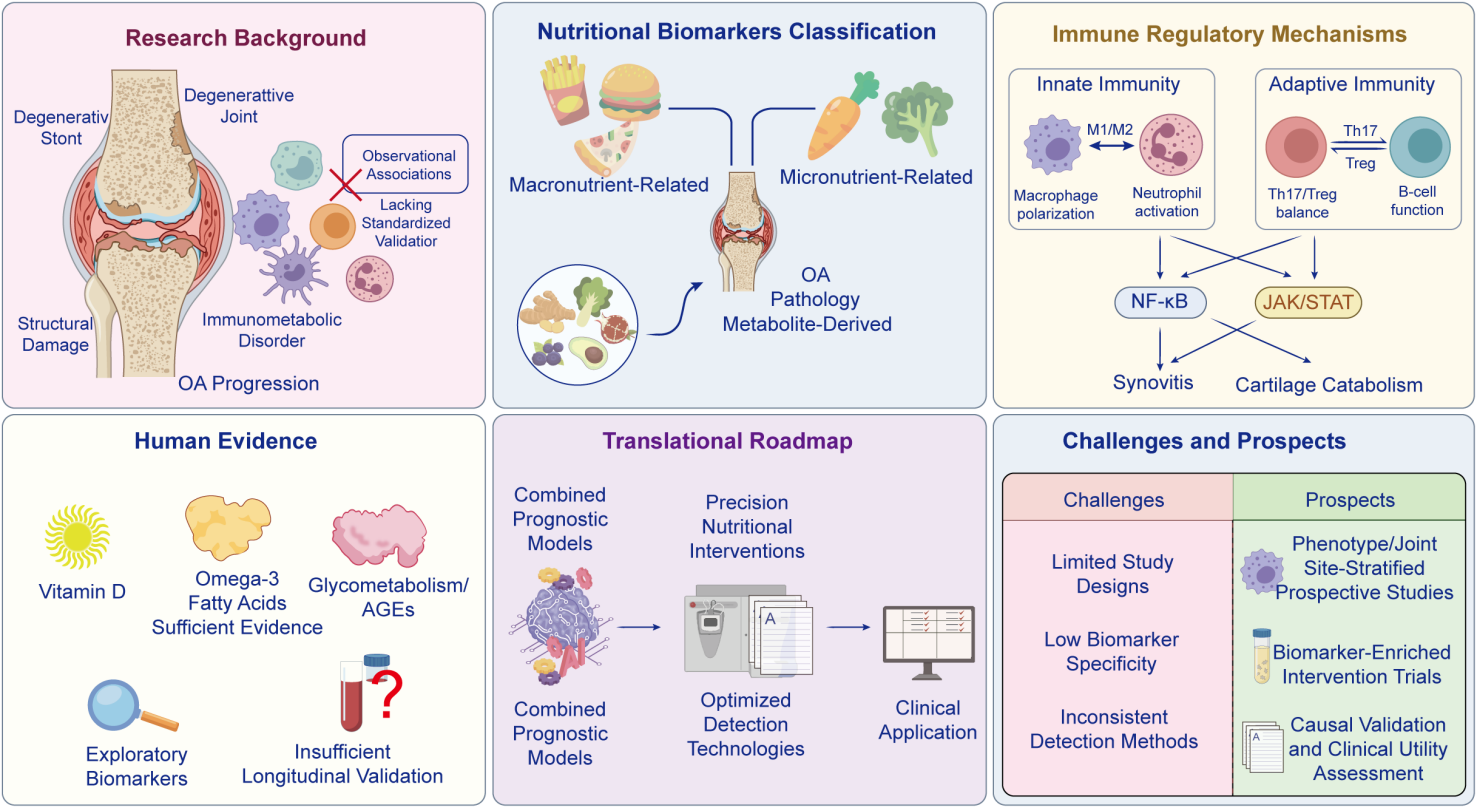
Immune-cell–driven synovitis and joint tissue destruction in inflammatory arthritis (illustrative immune microenvironment).

Joint pathology in OA emerges from bidirectional communication between immune cells and tissue-resident cells (chondrocytes, synovial fibroblasts/macrophages, osteoblasts, and osteoclasts). Nutritional biomarkers modulate this crosstalk by reshaping immune-cell cytokine outputs, redox tone, and lipid mediator production, thereby influencing cartilage matrix homeostasis and subchondral remodeling. The interaction between immune cells and joint tissue cells is shown in **Figure 6**.

Across the synovitis–cartilage–subchondral bone unit, inflammatory macrophage programs (IL-1β/TNF-α/IL-6) suppress anabolic SOX9 signaling and promote chondrocyte production of matrix-degrading enzymes, while osteoblast-derived TGF-β and Wnt ligands can drive aberrant angiogenesis and nerve ingrowth that couples structural remodeling to pain. Nutritional biomarkers linked to redox control (vitamin C/E status, selenium–GPX activity), lipid mediator balance (omega-6 eicosanoids *vs* omega-3-derived resolvins), or immunometabolic tone (SCFAs, glycemic/AGE measures) can therefore be mapped onto these cross-tissue feed-forward loops to provide OA-specific mechanistic interpretability.

Chondrocytes are the main functional cells of articular cartilage. Their metabolic abnormalities and functional damage are important pathological changes in OA. Pro-inflammatory factors secreted by immune cells can inhibit the proliferation of chondrocytes and the synthesis of collagen and proteoglycans, promoting the degradation of cartilage matrix (32). Nutritional biomarkers may help preserve chondrocyte function by shaping immune-chondrocyte signaling: vitamin D can act on chondrocytes and also restrain immune-cell cytokine production, thereby reducing inflammatory injury (32). Omega-3 fatty acids can reduce cartilage-matrix degradation by dampening pro-inflammatory lipid mediator signaling between immune cells and chondrocytes. Short-chain fatty acids may enhance chondrocyte stress resilience by modulating cellular metabolism and epigenetic programs; however, direct OA studies demonstrating that short-chain fatty acids reduce immune-cell cytotoxicity toward chondrocytes are currently lacking. Existing mechanistic support is derived mainly from broader gut–immune literature, animal models, and cell-based assays, often at concentrations that may exceed those achieved with typical nutritional intake, underscoring the need for joint-compartment measurements (e.g., synovial-fluid levels) and dose–response validation in OA-relevant systems.

Synoviocytes include synovial fibroblasts and synovial macrophages. After activation, they can secrete inflammatory factors and proteases, exacerbating joint inflammation and cartilage damage. Nutritional biomarkers may influence synovial inflammation by modulating redox tone and cytokine signaling within the synovium. For example, vitamin C has been reported to attenuate oxidative-stress–related inflammatory activation of synovial cells mainly in preclinical and *in vitro* contexts, but direct human OA evidence linking nutritional-range vitamin C status to reduced synovial inflammatory activity remains limited (32). Zinc can modulate synovial and immune-cell signaling in a dose-dependent manner, while selenium-related biomarkers may reflect antioxidant capacity that could buffer oxidative injury; however, for both trace elements, OA-specific mechanistic and interventional validation is still needed before strong regulatory claims can be made.

Bone cells include osteoblasts and osteoclasts. Abnormal functions of osteoblasts and osteoclasts can cause abnormal metabolism of subchondral bone, leading to abnormal stability of cartilage and remodeling of subchondral bone, which promotes the instability of subchondral bone and the development of OA. Cytokines secreted by immune cells can regulate the functions of osteoblasts and osteoclasts (35, 36). Nutritional biomarkers can regulate this process by regulating immune cells to secrete cytokines, thereby indirectly regulating the functions of osteoblasts and osteoclasts to maintain the stable balance of subchondral bone metabolism. Vitamin D can promote the proliferation and differentiation of osteoblasts and osteoclasts, inhibit osteoclast activity, and indirectly regulate the functions of osteoblasts and osteoclasts by regulating immune cells to secrete cytokines. Calcium and phosphorus can ensure the normal metabolism of osteoblasts and osteoclasts to maintain normal bone structure stability. Omega-3 fatty acids can reduce subchondral bone resorption caused by osteoclast activation and promote the functions of osteoblasts and osteoclasts, maintaining bone metabolism homeostasis (36).

Beyond structural progression, neuroimmune interactions provide an additional layer through which nutritional biomarkers may affect symptoms. Prostanoids derived from arachidonic acid (e.g., PGE2) and cytokines produced by activated macrophages and Th17 cells can sensitize peripheral nociceptors and promote central sensitization, whereas omega-3-derived pro-resolving mediators and antioxidant-associated biomarkers may attenuate inflammatory mediator production and oxidative stress that sustain pain pathways. This pain-relevant axis further supports organizing biomarker effects by pathological processes rather than by parallel biomarker lists.

This schematic contrasts a healthy joint with an inflamed joint characterized by synovial lining hyperplasia, increased vascular trafficking, immune-cell infiltration, cartilage damage, and bone erosion. In the healthy state, the articular cartilage surface remains intact, the synovial cavity is free of inflammatory infiltration, and subchondral bone architecture is preserved. In the inflamed joint, structural disruption is accompanied by synovial thickening and accumulation of infiltrating immune cells within the synovium and periarticular tissues. The enlarged panel illustrates an interactive inflammatory network involving macrophages, neutrophils, dendritic cells, monocytes, T cells, B cells, and synovial fibroblast-like cells. These cells communicate through a cytokine/chemokine molecular network and respond to damage-associated molecular patterns (DAMPs), including HMGB1 and S100A8/A9, via Toll-like receptor (TLR)-related signaling. This inflammatory circuitry promotes the production of pro-inflammatory mediators, such as TNF-α, IL-1β, IL-6, and GM-CSF, as well as matrix-degrading enzymes, including MMPs and ADAMTS, thereby driving persistent inflammatory activation, synovial hyperplasia, cartilage matrix breakdown, and progressive joint tissue destruction.

Abbreviations: HMGB1, high mobility group box 1; TLR, Toll-like receptor; TNF-α, tumor necrosis factor alpha; IL, interleukin; GM-CSF, granulocyte–macrophage colony-stimulating factor; MMPs, matrix metalloproteinases; ADAMTS, a disintegrin and metalloproteinase with thrombospondin motifs; DAMPs, damage-associated molecular patterns.

## 4 Human evidence and validation landscape for nutritional biomarkers in OA

From a translational perspective, nutritional biomarkers are best viewed as candidate indicators that reflect systemic immunometabolic states relevant to OA. At present, only a subset has been evaluated in sufficiently powered longitudinal cohorts or randomized interventions, and few studies have formally tested independence, calibration/discrimination, and external validity required for clinical risk prediction or monitoring. Accordingly, this section synthesizes the current validation landscape and highlights study designs and analytic practices needed to move from mechanistic plausibility to clinically actionable biomarkers.

In the context of risk stratification, prognostic interpretation requires alignment with standardized OA outcome definitions and transparent quantitative reporting. For risk prediction, studies should prespecify incident OA definitions (e.g., symptomatic OA based on validated criteria and/or incident radiographic OA defined by Kellgren–Lawrence grade) and report multivariable-adjusted effect estimates (OR/HR with 95% CI) per prespecified biomarker unit (per SD or clinically meaningful increment), alongside the full confounder set (age, sex, BMI/adiposity, prior injury, physical activity, comorbidities, and medication use).

With respect to disease monitoring and progression, biomarker utility is best evaluated using validated symptomatic and structural endpoints analyzed in appropriate longitudinal frameworks. Endpoints should include validated symptom instruments (e.g., WOMAC/KOOS) and structural outcomes (e.g., joint space width loss, MRI cartilage thickness loss and synovitis scores), analyzed using appropriate longitudinal models (mixed-effects/GEEs or time-to-event models for arthroplasty). When biomarkers are incorporated into prediction models, performance should be reported using discrimination (AUC/C-index), calibration (calibration plots/slope/intercept and Brier score), and external validation, with incremental value beyond clinical and imaging predictors.

Because many nutritional biomarkers are tightly coupled to adiposity, metabolic syndrome, overall diet quality, and systemic inflammatory burden, prognostic associations can be substantially confounded. Accordingly, studies claiming risk prediction or progression prediction should, at minimum, adjust for BMI (or direct adiposity measures), metabolic syndrome components (e.g., diabetes/insulin resistance and dyslipidemia), physical activity, prior joint injury, medication use, and baseline disease severity, and should evaluate phenotype-specific effects (e.g., metabolic *vs* synovitis-dominant OA) where possible.

Notably, most reports provide association estimates (OR/HR) rather than clinically deployable accuracy metrics; sensitivity/specificity and actionable cutoffs for nutritional biomarkers are rarely established and almost never externally validated. At present, no nutritional biomarker has consistently been shown to outperform conventional imaging markers (Kellgren–Lawrence grade, joint-space width, or MRI features), so future work should quantify incremental value beyond imaging using discrimination (AUC/C-index), calibration, reclassification, and decision-curve utility.

To facilitate translational prioritization, we summarize nutritional biomarkers with comparatively stronger clinical potential, including their direction of change in OA, dominant immunomodulatory mechanisms, and representative human evidence relevant to OA risk prediction, disease monitoring/progression, and treatment response (**Table 2** and **Table 3**).

**Table 2:** Clinically promising nutritional biomarkers in osteoarthritis: direction of change, immunomodulatory mechanisms, and prognosis-related evidence.

Nutritional biomarker (sample/assay)	Typical trend in OA	Main immunomodulatory mechanisms (immune targets and pathways)	Prognostic/clinical evidence in OA (risk/progression/response)
Vitamin D status (serum 25[OH]D; 1,25[OH]2D3)	↓; lower levels often associate with higher symptom and/or structural burden	VDR signaling restrains pro-inflammatory macrophage programs, promotes tolerogenic DC phenotypes, suppresses Th17 polarization and supports Treg function; dampens NF-κB and cytokine amplification via JAK/STAT.	Observational evidence links deficiency with increased OA risk and worse clinical outcomes; supplementation studies suggest potential benefit in subsets with baseline deficiency, supporting use for stratification and response monitoring. Recent retrospective clinic-based evidence reinforces the high prevalence of deficiency in knee OA and its association with bilateral involvement and polyarthralgia, although routine inflammatory indices were not consistently correlated (14).
Serum albumin/prealbumin	↓ with chronic inflammation and catabolic states	Integrative marker of nutritional and inflammatory status; low levels reflect impaired immunometabolic fitness and a systemic pro-inflammatory milieu that can potentiate synovitis.	Associations with disease vulnerability and poorer overall prognosis (functional status and recovery capacity) support inclusion in multivariable models rather than use as a stand-alone OA biomarker. A retrospective cross-sectional study reported stepwise decreases in albumin and the Prognostic Nutritional Index with increasing radiographic severity and proposed cutoffs to discriminate stages, supporting its potential role in progression stratification pending prospective validation (7).
Omega-3 PUFAs (EPA/DHA; omega-3 index)/pro-resolving mediators	↓ and/or low omega-3 intake; normalization with supplementation	PPARγ activation and specialized pro-resolving mediator signaling promote macrophage M2-like states, reduce neutrophil recruitment, and attenuate NF-κB/MAPK-driven cytokine production.	Dietary/serum measures correlate with inflammatory phenotype and symptom severity; intervention evidence supports symptomatic improvement in some cohorts, suggesting utility for patient stratification. However, in a large prospective cohort at risk for knee OA, baseline fasting serum levels of specific n-3 fatty acids (including EPA) were not associated with incident OA or other OA outcomes, highlighting heterogeneity across formulations, biomarkers, and populations (9).
Omega-6–derived eicosanoids (e.g., PGE2, leukotrienes)/omega-6:omega-3 imbalance	↑ pro-inflammatory lipid mediators; ↑ omega-6:omega-3 ratio	COX/LOX–dependent lipid mediator production amplifies synovitis via EP/leukotriene receptors, reinforces pro-inflammatory macrophage programming, and contributes to nociceptor sensitization.	Higher pro-inflammatory lipid signatures associate with synovitis activity and pain; may serve as a mechanistic readout for anti-inflammatory dietary or pharmacologic interventions.
Short-chain fatty acids (acetate/propionate/butyrate)	Often ↓ in dysbiosis-associated metabolic states	GPR41/43 signaling and HDAC inhibition promote Treg differentiation, restrain inflammatory macrophage metabolism, and can suppress inflammasome-related IL-1β production; reflects gut–joint immune crosstalk.	Lower levels associate with worse pain/inflammation in observational studies; promising for monitoring microbiome- or fiber-based interventions, though assay standardization is required. Caveat: OA-specific longitudinal cohorts and externally validated prognostic models for SCFAs are limited, and sensitivity/specificity cutoffs are not established; thus SCFAs are best considered exploratory markers for microbiome/fiber-related immunometabolic states and intervention monitoring.
Glycemic/AGE-related markers (fasting glucose, HbA1c, AGEs)	↑ in metabolic OA phenotypes; higher AGE burden with severity	AGE–RAGE signaling drives NF-κB activation and oxidative stress, enhances pro-inflammatory macrophage responses, and facilitates NLRP3 inflammasome activation with IL-1β release; promotes chondrocyte catabolism.	Consistent associations with OA progression and symptom burden in metabolic comorbidity settings; supports risk stratification and prognosis in ‘metabolic OA’ and as a modifiable target through glycemic control.
Adipokines (leptin, adiponectin; leptin/adiponectin ratio)	Leptin ↑; adiponectin variable; ratio often ↑	Leptin enhances macrophage and T-cell pro-inflammatory signaling (including JAK/STAT and NF-κB), increasing cytokine production and linking adiposity to synovitis; adiponectin may counter-regulate inflammation depending on context.	Associated with obesity-related OA phenotypes, pain, and inflammatory burden; may inform prognosis and response to weight-loss/nutritional interventions when interpreted alongside BMI and metabolic status.
Selenium status (serum selenium; GPx activity)	Often ↓; deficiency linked to higher oxidative stress	Supports antioxidant selenoproteins that limit ROS-driven amplification of NF-κB/MAPK signaling and preserve immune-cell homeostasis; may reduce oxidative cartilage injury.	Lower status correlates with worse symptoms in some cohorts; translational potential as an adjunctive biomarker, but requires prospective validation and careful dose considerations. Caveat: existing findings are largely observational with heterogeneous assays and incomplete confounder control (BMI/metabolic health, diet, supplementation). Prospective validation and dose-aware analyses are needed before selenium is claimed as a stand-alone prognostic marker.
Zinc status (serum/plasma zinc; Zn/Cu ratio)	Variable; deficiency or imbalance reported in some cohorts	Supports immune-cell development and antioxidant enzymes (e.g., Zn-dependent SOD); influences cytokine signaling and may modulate MMP activity indirectly via inflammatory tone.	Human OA evidence is limited and inconsistent, and levels are influenced by diet, inflammation, and comorbidities.
Uric acid/urate-related metabolites	↑ in hyperuricemia; episodic increases may parallel inflammatory flares	Urate crystals can activate innate immune sensing and the NLRP3 inflammasome, promoting IL-1β–mediated synovitis and pain; intersects with metabolic inflammation.	Higher levels associate with inflammatory exacerbations and symptom burden, especially with gout/metabolic comorbidity; may support phenotype classification and targeted management.

**Table 3:** Representative human studies evaluating candidate nutritional biomarkers in OA with multivariable modeling and/or randomized designs.

Biomarker (specimen)	Human evidence (design/population)	OA endpoint	Analysis/modeling	Main finding & caveats
Diabetes/hyperglycemia (glucose, HbA1c; serum)	Nationwide cohort (glycemic status) (37); MR HbA1c (38)	Incident symptomatic KOA	Multivariable Cox; MR	Higher glycemia linked to higher KOA risk; phenotype heterogeneity/confounding remain.
25(OH)D (serum)	VIDEO RCT *post hoc* effect modification (39); supportive evidence (40, 41). Additional retrospective evidence in knee OA supports high deficiency prevalence and links more severe deficiency to bilateral disease and polyarthralgia, although routine inflammatory indices were not consistently associated (14).	Pain/function; cartilage loss; synovitis	RCT; interaction/subgroup	Baseline vitamin D may stratify response; *post hoc* findings need prospective confirmation.
Omega-3 PUFA signatures/supplementation	Meta-analysis of RCTs in OA (42)	Pain; function	RCT meta-analysis	Modest benefit overall; dose/formulation and diet background vary.
Krill oil (omega-3-rich supplement)	Randomized placebo-controlled trial in inflammatory knee OA (43)	Pain (24 weeks)	RCT (ITT)	No analgesic benefit *vs* placebo; underscores need for biomarker-enriched phenotypes.
Skin AGEs (autofluorescence)	Population study with radiographic OA and MRI outcomes (44)	OA severity; cartilage loss	Adjusted regression	Associations observed in some strata; cross-sectional elements limit causality.
Adipokines (leptin, adipsin; serum)	Symptomatic knee OA cohort; MRI progression/TKR (45)	Progression; total knee replacement	Longitudinal regression	Higher adipokines linked to progression/TKR; adiposity mediation likely.
Metabolic syndrome components	UK Biobank prospective cohort (46)	Incident OA	Adjusted hazard models	MetS associates with OA risk; specificity limited; must test incremental value beyond BMI.
Serum albumin/Prognostic Nutritional Index (PNI; serum)	Retrospective cross-sectional study in gonarthrosis with severity stratification and ROC analyses (7)	Radiographic severity (Kellgren–Lawrence stage) and stage discrimination	Albumin and PNI decreased stepwise with greater radiographic severity; proposed cutoffs discriminated early *vs* late stage, but prospective progression validation is needed (7).	–
Specific n-3 and n-6 essential fatty acids (serum; fasting)	Prospective cohort (MOST) with baseline fasting assays and multivariable logistic regression through 60 months (9)	Incident symptomatic and radiographic knee OA; MRI cartilage/synovitis; pain outcomes	No association of EPA or other specific n-3/n-6 fatty acids with incident OA or other OA outcomes after multivariable adjustment, arguing against simple causal inference from circulating FA levels (9).	–
Standard lipid biomarkers (HDL, LDL, triglycerides; serum)	NHANES multivariable logistic regression complemented by two-sample Mendelian randomization (47)	Prevalent OA (NHANES) and causal inference proxy via GWAS-based MR	Observed associations were small; MR suggested a modest protective association for LDL while HDL required further study, highlighting the need for imaging-defined OA outcomes and careful confounder control (47).	–

Note: Direction of change may vary by joint site, OA phenotype, diet, and comorbidities; evidence strength is currently highest for vitamin D status, omega-3-related lipid signatures, and metabolic/AGE-related measures, whereas other biomarkers require prospective standardization and validation. In this context, apparent biomarker performance may largely reflect confounded correlations with BMI/metabolic health or baseline imaging severity rather than OA-specific biology, underscoring the need for rigorous confounder control and external validation.

Importantly, the term “prognostic value” should be reserved for biomarkers that have been validated in appropriately designed epidemiological studies and/or clinical trials, where their independent associations with OA outcomes are quantified using multivariable regression (e.g., logistic regression for incident symptomatic/radiographic OA, Cox models for time-to-event outcomes such as joint replacement, and mixed-effects models for longitudinal pain or imaging trajectories). For clinical deployment, studies should report adjusted effect estimates, assess model discrimination and calibration, and demonstrate incremental value beyond established predictors, following transparent reporting standards for prediction model development and validation (5, 48).

From an evidence-based perspective, most prognosis-related findings remain associative, derived from cross-sectional analyses or modest-sized cohorts with variable follow-up and incomplete adjustment for key confounders. Few studies quantify incremental predictive value beyond established clinical and imaging predictors (e.g., age, BMI, Kellgren-Lawrence grade) or report external validation. For clinical utility, future studies should predefine sampling conditions (fasting state, timing), harmonize assays and cutoffs, and evaluate biomarker panels using calibration, discrimination, and decision-curve analyses in longitudinal, phenotype-stratified cohorts and randomized nutritional interventions.

Encouragingly, regression-based evidence is emerging for selected nutritional biomarkers. For instance, a nationwide cohort study reported that diabetes status and hyperglycemia were associated with symptomatic knee OA using time-to-event regression with multivariable adjustment (37). Complementary genetic evidence from a two-sample Mendelian randomization study supported a causal effect of genetically higher HbA1c on knee OA risk (38). In the interventional setting, a *post hoc* analysis of the VIDEO randomized trial suggested that baseline 25-hydroxyvitamin D levels may modify the symptomatic and structural response to vitamin D supplementation (39). In the lipid domain, a recent meta-analysis of randomized trials reported modest improvements in pain and function with omega-3 polyunsaturated fatty acid supplementation (42), whereas a large placebo-controlled trial found no analgesic benefit of krill oil in an inflammatory knee OA phenotype (43). These examples highlight both feasibility and heterogeneity, reinforcing the need for phenotype-aware, model-based validation when proposing prognostic or predictive nutritional biomarkers.

### 4.1 Risk association and incident OA

Evidence linking nutritional biomarkers to incident OA is still dominated by observational designs; thus, associations should be interpreted with careful adjustment for confounding (e.g., BMI, physical activity, comorbidities) and, ideally, replicated in independent prospective cohorts. Nutritional biomarkers can be used as predictors of the risk of OA, helping identify high-risk populations and implement early disease prevention. Vitamin D deficiency is a known risk factor for OA. A decrease in serum 25-hydroxyvitamin D levels is significantly associated with an increased risk of OA, especially knee and hip OA. Low levels of vitamin D can cause immune dysfunction (29, 49), enhanced inflammatory responses, impaired chondrocyte metabolism, and reduced stability of subchondral bone, thereby increasing the risk of disease. Notably, vitamin D–OA associations reported in observational studies are not always consistent after adjustment for adiposity and physical activity; therefore, standardized cutoffs and external validation in multivariable prediction models are required before vitamin D status can be used for individualized risk prediction (5, 48). A recent retrospective study of 986 patients with knee OA reported a high prevalence of vitamin D deficiency and found that more severe deficiency was associated with bilateral disease and polyarthralgia, although it was not significantly associated with routine low-grade inflammatory indices (ESR and platelet count), underscoring phenotype dependence and the need for longitudinal validation (14). Where candidate biomarkers are proposed for risk prediction, analyses should report multivariable-adjusted ORs/HRs (95% CI) using standardized outcome definitions, and prediction models should additionally provide discrimination (AUC/C-index), calibration, and external validation to demonstrate generalizability. Consequently, reliable sensitivity/specificity estimates for individual nutritional biomarkers in OA risk prediction are generally not available, because most studies do not report standardized cutoffs or validate ROC-based thresholds across independent cohorts.

Albumin is an important indicator reflecting the body’s protein nutritional status and systemic inflammatory state. A decrease in serum albumin levels is associated with an increased risk of OA. Hypoalbuminemia can lead to insufficient energy supply for immune cells, resulting in dysfunction, aggravating inflammatory responses and joint tissue damage, and increasing the possibility of OA. The imbalance between Omega-3 fatty acids and Omega-6 fatty acids is also a risk factor for OA (8, 50). A decrease in serum Omega-3 fatty acid levels or an increase in Omega-6 fatty acid levels can lead to enhanced inflammatory responses and an increased risk of disease. For omega-3–related signatures, randomized evidence suggests small-to-moderate symptomatic benefits overall, but effects vary by formulation, dose, and inflammatory phenotype, as illustrated by recent trial-level syntheses and a large krill oil trial in knee OA (42, 43). Importantly, prospective evidence does not uniformly support a protective role of circulating essential fatty acids: in the Multicenter Osteoarthritis Study (MOST), baseline fasting serum levels of specific n-3 (including EPA) or n-6 fatty acids were not associated with incident symptomatic or radiographic knee OA, nor with other OA outcomes over 60 months after multivariable adjustment (98). Beyond fatty-acid profiles, a combined NHANES analysis with multivariable logistic regression and a complementary two-sample Mendelian randomization analysis suggested only small associations between standard lipid markers (HDL/LDL/TG) and OA, with LDL showing a modest protective signal in MR; these findings warrant replication using imaging-defined phenotypes and rigorous confounder control (47).

In addition, abnormal levels of nutritional biomarkers such as short-chain fatty acids, zinc, selenium, and vitamin C are also related to the risk of OA. By combining the detection of these nutritional biomarker levels with clinical indicators such as age, gender, body mass index, and joint injury history, a prediction model for the risk of OA can be constructed to improve the identification efficiency of high-risk OA populations and facilitate early intervention. However, for SCFAs, selenium, and zinc, the current OA literature is largely observational and often lacks comprehensive adjustment for BMI/adiposity, metabolic syndrome, and physical activity; therefore these markers should be considered exploratory risk indicators until supported by well-powered longitudinal cohorts and externally validated prediction models.

Subtype- and phenotype-specific evidence for disease risk prediction. OA is clinically and biologically heterogeneous across joint sites (knee, hip, hand) and etiologic phenotypes (post-traumatic, metabolic/obesity-related, age-related, and synovitis-dominant). Accordingly, the direction and magnitude of associations for nutritional biomarkers can differ by subtype, and candidates that perform well in one context may not generalize to others; therefore, risk-prediction analyses should prespecify joint site and phenotype, and report interaction testing where appropriate.

At present, most regression-based and interventional biomarker evidence is derived from knee OA cohorts. For example, vitamin D deficiency has been linked to knee OA burden in recent clinic-based data (14), and serum albumin/PNI showed stepwise decreases with increasing radiographic severity in gonarthrosis (7). In contrast, in the Multicenter Osteoarthritis Study (MOST), baseline fasting serum levels of specific n-3 or n-6 fatty acids (including EPA) were not associated with incident symptomatic or radiographic knee OA or other outcomes after multivariable adjustment (9), and a placebo-controlled trial reported no analgesic benefit of krill oil in an inflammatory knee OA phenotype (43). Metabolic biomarkers (e.g., hyperglycemia/HbA1c and AGE-linked measures) may be more informative for metabolic OA, supported by prospective and genetic evidence relating dysglycemia to knee OA risk (37, 38). By comparison, subtype-specific validation for hip and hand OA remains limited, underscoring the need for adequately powered, site-stratified cohorts and external validation before clinical translation.

### 4.2 Longitudinal progression and structural outcomes

Longitudinal evidence for biomarker association with pain trajectories and structural progression remains heterogeneous, often limited by modest sample size, variable imaging endpoints, and incomplete accounting for baseline disease severity. Nutritional biomarkers can reflect the severity and progression of OA, providing objective basis for disease assessment. Serum vitamin D levels are negatively correlated with the severity of OA. Patients with more severe conditions have lower serum vitamin D levels. Low levels of vitamin D can promote disease progression by exacerbating immune-inflammatory responses and cartilage damage, while vitamin D supplementation can delay disease progression (14). In addition to cross-sectional severity correlations, *post hoc* analyses of randomized supplementation trials suggest that baseline vitamin D status may influence cartilage loss trajectories and symptom improvement, supporting a stratified approach when evaluating vitamin D as a progression-related biomarker (39). For progression claims, studies should prespecify symptom endpoints (e.g., WOMAC/KOOS trajectories) and structural outcomes (e.g., joint-space width loss thresholds, MRI cartilage thickness or synovitis scores), report adjusted longitudinal effect estimates (beta coefficients or HRs with 95% CI), and account for baseline disease severity and time-varying treatments to limit bias.

Serum albumin levels can reflect the overall nutritional status and inflammatory level of patients. With the development of OA, chronic inflammation intensifies, and serum albumin decreases. Changes in serum albumin levels can be used as a criterion for judging disease progression. Short-chain fatty acids are decomposition products of dietary fiber. Serum short-chain fatty acid levels are negatively correlated with the degree of joint pain and swelling in OA patients (7, 28). Lower serum levels indicate more severe conditions, and their changes can judge the trend of disease progression. In a recent retrospective cross-sectional study, serum albumin and the Prognostic Nutritional Index (PNI) decreased stepwise with higher Kellgren–Lawrence stage, and albumin/PNI thresholds showed diagnostic performance for distinguishing early- versus late-stage gonarthrosis and for identifying patients at risk of late-stage disease; nevertheless, the cross-sectional design precludes causal inference and prospective progression validation remains necessary (7). These observations are derived mainly from small cross-sectional datasets and indirect gut–joint axis studies, with limited standardization of specimen (stool *vs* serum), fasting status, and analytical platforms; robust longitudinal cohort evidence demonstrating baseline SCFAs as independent predictors of incident OA, structural progression, or treatment outcomes is currently sparse. Therefore, SCFAs should be framed as candidate markers of microbiome-related immunometabolic state, and their clinical sensitivity/specificity for OA prognosis remains to be established in externally validated models.

In addition, the decrease in levels of micronutrient-related biomarkers such as zinc, selenium, and vitamin C, as well as the imbalance between pro-inflammatory and anti-inflammatory factors, are related to the progression of OA. Dynamic monitoring of changes in relevant nutritional biomarker levels can timely and dynamically assess the patient’s disease progression and adjust the treatment plan (51). Nevertheless, for trace elements (selenium and zinc) and antioxidant vitamins, OA-specific longitudinal evidence is inconsistent and often susceptible to confounding by diet quality, supplement use, comorbid inflammation, and adiposity; in addition, the biologically relevant window may differ between nutritional sufficiency and pharmacologic supplementation, so prognostic interpretation should be restricted to studies with standardized assays, predefined cutoffs, and multivariable longitudinal modeling.

### 4.3 Intervention studies and treatment response

For treatment-response applications, randomized trials with prespecified biomarker analyses and standardized sampling are required; *post hoc* subgroup findings should be treated as hypothesis-generating until externally replicated. Nutritional biomarkers can be used to track and monitor the effectiveness of OA patients during treatment, providing reference for adjusting medications and formulating treatment plans. Currently, the main treatments for OA include drug therapy, physical therapy, and surgical therapy. Nutritional intervention, as a supplementary measure, has gradually gained attention, and changes in nutritional biomarker levels can be used to evaluate treatment effects and whether the patient’s nutritional status has improved (52). When biomarkers are used to predict therapeutic response, trials should apply standardized responder definitions (e.g., OMERACT–OARSI criteria or prespecified changes in WOMAC/KOOS), formally test biomarker-by-treatment interactions, and report effect sizes and model performance metrics (AUC/C-index and calibration) with independent validation whenever feasible.

In patients receiving nutritional intervention, increased levels of biomarkers such as serum vitamin D, Omega-3 fatty acids, and short-chain fatty acids mostly reflect effective intervention, indicating that their immune-inflammatory responses are inhibited and the condition is relieved. For example, after vitamin D supplementation, patients’ serum 25-hydroxyvitamin D levels increase, joint pain and swelling symptoms are alleviated, and inflammatory factor levels decrease, indicating a good treatment response (14). Nevertheless, recent high-quality trials indicate that supplementation-related biomarker changes do not necessarily translate into symptomatic benefit; for example, krill oil did not improve knee pain over 24 weeks despite its anti-inflammatory rationale in a multicenter randomized, placebo-controlled trial. Conversely, in the VIDEO trial, baseline 25-hydroxyvitamin D appeared to predict differential symptomatic and structural responses to vitamin D supplementation, highlighting the value of biomarker-guided enrichment and interaction testing (39).

For patients receiving drug therapy, changes in nutritional biomarker levels reflect the drug treatment effect and patient tolerance. For example, non-steroidal anti-inflammatory drugs can resolve joint inflammation, but long-term use can affect gastrointestinal function and nutrient absorption. A decrease in levels of biomarkers such as serum albumin and vitamins suggests the need to adjust drug dosage or strengthen nutritional support.

At the same time, nutritional biomarkers can also be used to predict patients’ treatment responses to assist in the selection of personalized treatment. For example, patients with higher serum vitamin D levels may have a better response to immunomodulatory therapy, while patients with lower serum Omega-3 fatty acid levels may be suitable for nutritional intervention with Omega-3 fatty acid supplementation. However, these predictive statements should be interpreted cautiously unless supported by prospective trials that formally test biomarker–treatment interactions (e.g., regression models with interaction terms) and provide external validation across cohorts and OA phenotypes (5, 48).

## 5 Translational roadmap and study design considerations

Building on mechanistic insights, the translational value of nutritional biomarkers in OA will depend on rigorous clinical validation and standardized implementation pathways, rather than descriptive associations alone. We therefore outline key design and reporting considerations to enable reproducible risk/monitoring models and biomarker-guided nutritional interventions.

### 5.1 Developing and validating risk/monitoring models

Candidate nutritional biomarkers can be integrated with established clinical variables (e.g., age, sex, BMI, prior injury), imaging metrics, and molecular biomarkers to develop risk prediction or monitoring models. However, model development should be anchored to prespecified endpoints, sufficient events-per-variable, and transparent reporting of adjusted effect estimates and performance metrics (discrimination, calibration) with internal and external validation. For reproducibility, model development should clearly define outcomes and time horizons, describe biomarker preprocessing (units, transformations, batch effects), prespecify the adjustment set, and report missing-data handling. Internal validation (bootstrapping/cross-validation) and external validation should be accompanied by transparent quantitative reporting, including adjusted OR/HR estimates, discrimination (AUC/C-index), calibration (plots and slope/intercept; Brier score), and clinical utility (decision-curve analysis or net benefit).

In addition to statistical significance, models should demonstrate incremental predictive value beyond conventional predictors (e.g., change in C-statistic/AUC, net reclassification improvement) and decision-analytic utility to justify clinical use. Where feasible, biomarker panels should be evaluated in prospective cohorts and embedded within randomized nutritional or pharmacologic trials to establish clinical actionability.

### 5.2 Constructing combined prognostic models

Nutritional biomarkers can be combined with clinical indicators, imaging indicators, and other biomarkers (inflammatory factors, cartilage degradation products) to construct combined prognostic assessment models for OA, improving prediction accuracy and reliability. For example, combining nutritional biomarkers such as serum vitamin D, albumin, and short-chain fatty acids with indicators such as age (8, 51), gender, body mass index, joint pain score, and X-ray Kellgren-Lawrence classification to construct a prediction model for the risk of OA, which is used for screening high-risk populations. Combining nutritional biomarkers with inflammatory factors (tumor necrosis factor-α, interleukin-6) and cartilage degradation products (matrix metalloproteinase-3, hyaluronic acid) to construct a disease progression prediction model for evaluating the severity and development dynamics of patients’ conditions (43, 53–55).

Methodologically, combined prognostic models are commonly constructed using multivariable logistic regression for binary outcomes (e.g., incident OA, radiographic/pain progression) and Cox proportional hazards models for time-to-event endpoints (e.g., arthroplasty). Continuous biomarkers are preferably modeled without arbitrary dichotomization, with non-linear terms (e.g., splines) and pre-specified interactions to reflect OA phenotypes. To mitigate overfitting when multiple candidate biomarkers are evaluated, penalized regression (LASSO/elastic net) or shrinkage with bootstrap/cross-validation can be applied, followed by internal and external validation reporting discrimination (AUC/C-index), calibration, and, where relevant, decision-curve net benefit.

Notably, recent OA biomarker consortia provide concrete precedents for such multivariable pipelines. In the FNIH Biomarkers Consortium PROGRESS OA study, elastic-net logistic regression identified a combined model including serum hyaluronan and urinary C2C-HUSA together with BMI and Kellgren-Lawrence grade, yielding an AUC of 0.627 for predicting clinically relevant joint-space width loss (≥0.7 mm) over 12-36 months (56). Similarly, Zhou et al. developed an MRM-based serum proteomic panel (15 markers/13 proteins) and demonstrated improved discrimination for clinically relevant progression (structural + pain) relative to covariates alone (AUC ~0.73 in the FNIH cohort), with independent validation in the Biomarker Factory cohort (57). These examples support the feasibility of extending analogous modeling frameworks to nutritional biomarker panels, provided that endpoints and confounders are rigorously defined and models are externally validated.

Therefore, combined prognostic models can provide an effective tool for better identifying high-risk populations, assessing patient conditions, and predicting patient prognosis in clinical practice, providing scientific guidance for personalized clinical treatment plans. For example, early nutritional intervention and immunomodulatory therapy can be adopted for high-risk patients to delay the onset of the disease (43, 53–55), and necessary intensive treatment can be taken for patients with rapid disease progression to prevent further deterioration of joint damage.

### 5.3 Biomarker-informed nutritional intervention strategies

Mechanism-guided nutritional interventions should be evaluated in adequately powered randomized trials with prespecified biomarker stratification and longitudinal sampling, so that biomarker changes can be interpreted as intermediate outcomes and/or predictors of clinical benefit. In this context, biomarker measurements (e.g., serum 25(OH)D, omega-3 lipid signatures, or SCFA profiles) are endpoints for stratification and monitoring, whereas dietary intake patterns and supplementation are exposures/interventions intended to modify these endpoints (3, 27). Accordingly, future studies should enrich enrollment based on baseline deficiency or adverse biomarker profiles, formally test biomarker-treatment interactions using multivariable models, and link achieved biomarker targets to symptom and imaging outcomes within predefined OA phenotypes.

When formulating personalized nutritional intervention strategies, they can also be developed according to the patient’s comorbidities (such as diabetes, hypertension, obesity). For example, OA patients with diabetes need to control postprandial blood glucose levels (23), and their diet should be low in sugar and high in dietary fiber. Obese patients need to ensure sufficient energy intake without excess to avoid nutritional deficiencies caused by weight loss.

### 5.4 Optimizing clinical detection technologies

Currently, the main methods for detecting nutritional biomarkers include serum detection, urine detection, and tissue biopsy, which have certain limitations. For example, tissue biopsy is invasive, and the sensitivity and specificity of some serum biomarkers are still problematic. In the future, the precision (58, 59), sensitivity, and simplicity of detecting nutritional biomarkers can be improved by improving detection methods, promoting the clinical application of nutritional biomarker detection.

For example, emerging analytical approaches—including targeted LC–MS/MS assays for short-chain fatty acids in plasma and fecal matrices—can improve sensitivity and reproducibility for low-abundance metabolites and may be adapted for OA-relevant nutritional readouts (60, 61). Non-invasive or minimally invasive sampling (e.g., feces and saliva, and capillary blood microsampling) could facilitate longitudinal monitoring, but requires strict control of pre-analytic variability and matrix effects (62–64). Laboratory automation and robotic sample preparation can increase throughput and reduce operator-dependent variability in LC–MS–based workflows; however, whether such automation meaningfully improves clinical decision-making for OA (risk stratification, progression monitoring, or treatment-response assessment) remains to be demonstrated in prospective OA cohorts with analytical validation, cross-platform harmonization, and cost-effectiveness assessment (65).

In addition, standards and reference values for nutritional biomarker detection methods can be established, unifying standard operating procedures and detection processes to ensure the reliability and comparability of detection results, providing a unified application standard for clinical practice.

## 6 Challenges and prospects

### 6.1 Critical appraisal of the current evidence base

- Study design and causality: The literature is largely cross-sectional or case-control, limiting causal inference. Prospective cohorts are fewer, and randomized trials are often heterogeneous in baseline nutritional status, dose, duration, and endpoints, resulting in variable effect estimates.

- Phenotypic and anatomical heterogeneity: OA is biologically diverse (metabolic, post-traumatic, age-related phenotypes; knee *vs*. hip *vs*. hand), yet many studies pool phenotypes, which can dilute biomarker-specific signals and reduce reproducibility.

- Confounding and reverse causation: Body composition, comorbidities (diabetes, CKD, cardiovascular disease), medications (e.g., NSAIDs, statins), supplement use, and physical activity can influence both biomarkers and OA outcomes. Pain-related dietary changes and reduced mobility may further bias associations.

- Biomarker measurement limitations: Single-time systemic measurements may not reflect the intra-articular immune microenvironment. Pre-analytical factors (fasting, circadian variation, storage), assay platforms, and lack of standardized cutoffs contribute to between-study inconsistency.

- Mechanistic extrapolation: Many immunomodulatory pathways are inferred from *in vitro*/animal studies or other inflammatory conditions; direct human synovial validation and mediation analyses linking biomarker changes to immune-cell reprogramming and structural outcomes remain limited.

- Evidence synthesis and reporting: Publication bias and insufficient reporting of effect sizes and model performance (discrimination, calibration, net benefit) hinder objective comparison and clinical prioritization.

Although research on nutritional biomarkers in OA has yielded mechanistic insights into immune regulation, the current human evidence for clinical validation remains uneven and, for many candidates, insufficient for routine prognostic or monitoring use.

The specificity of nutritional biomarkers needs to be improved. Most of the currently discovered nutritional biomarkers are not only related to OA but also interfered by other diseases (such as cardiovascular diseases, diabetes, chronic kidney diseases) and lifestyles (such as smoking, drinking, exercise). Their low specificity in OA limits their application as prognostic and diagnostic biomarkers. The causal relationship between nutritional biomarkers and OA is not clear. Most current studies are cross-sectional or cohort studies, which have confirmed the correlation between nutritional biomarkers and OA, but cannot determine whether there is a causal relationship between them. More intervention studies and animal experiments are needed to verify this. The immune mechanisms by which nutritional biomarkers regulate OA need to be further revealed. Current research on the intervention of nutritional biomarkers on OA immunity is mostly carried out at the cellular and molecular levels. The specific signal transduction, molecular targets, and cellular network mechanisms still need to be explored (53–55, 66). Clinical translation and application are difficult. The detection methods of nutritional biomarkers are not yet mature, and standard operating procedures and reference data have not been unified. There is a lack of standards for clinical diagnosis and treatment guided by nutritional biomarkers, which is not convenient for clinical practical application. At the same time, the effect of personalized nutritional intervention is also affected by factors such as the compliance of intervention objects, differences in intestinal flora, and genetic background, which need to be improved urgently.

### 6.2 Priorities to strengthen evidence and enable translation

To move from descriptive associations toward clinically actionable biomarkers, future work should adopt rigorous study designs, standardized measurement, and transparent reporting.

We added OA-specific, design-oriented priorities to address key gaps in specificity, causality, and clinical utility:

- Phenotype- and joint site-stratified prospective cohorts: enroll knee/hip/hand OA separately and prespecify endotypes (e.g., metabolic *vs* synovitis-dominant) using baseline imaging and inflammatory readouts; collect repeated biospecimens (fasting blood, urine, and stool) and outcomes (WOMAC/KOOS, radiographs, and MRI cartilage/synovitis) at fixed intervals (e.g., 12-24 months) to enable time-varying and progression analyses.

- Joint-compartment linkage substudies: in a nested subset, obtain paired serum and synovial-fluid (and, when clinically indicated, synovial tissue) samples to quantify local metabolites (including SCFAs and lipid mediators) and immune-cell programs (single-cell or spatial profiling). These data enable mediation analyses testing whether systemic biomarkers track intra-articular immune reprogramming and structural change.

- Dose-aware mechanistic experiments: complement human cohorts with ex vivo human synovium-cartilage co-cultures and OA-relevant animal models using physiologic (nutritional-range) concentrations when possible, and explicitly report when pharmacologic dosing is required to achieve an effect. This improves interpretability and reduces over-extrapolation from supra-physiologic *in vitro* conditions.

- Biomarker-enriched randomized trials: test causality using stratified designs (e.g., vitamin D supplementation restricted to deficiency; omega-3 interventions in participants with low omega-3 index; fiber/prebiotic interventions in low-SCFA or dysbiosis signatures). Prespecify biomarker-by-treatment interactions, verify exposure by serial biomarker measurements, and include structural endpoints (MRI/cartilage thickness, synovitis) alongside symptoms.

- Confounder measurement and control: measure adiposity (BMI plus waist or body composition), metabolic syndrome components, medication and supplement use, and physical activity (preferably accelerometry) to reduce residual confounding; incorporate diet assessment with repeated recalls/FFQs to distinguish intake from circulating status and to model measurement error.

- Clinical utility benchmarking: evaluate whether nutritional biomarkers add incremental value beyond established predictors by comparing models with and without imaging markers (KL grade, joint-space width, MRI features), reporting discrimination, calibration, and decision-curve utility, and performing external validation across independent cohorts.

Nutritional biomarkers may contribute to OA precision medicine by linking immunometabolic states to symptom and structural trajectories; however, realizing this potential requires rigorously designed longitudinal cohorts and biomarker-stratified trials that demonstrate independent and incremental clinical utility.

## 7 Conclusion

In conclusion, immunonutritional biomarkers provide a pragmatic window into systemic immunometabolic states that may interact with OA-relevant immune circuits and tissue stress responses, particularly in inflammation- and metabolic-stress-enriched phenotypes. Rather than acting as disease-specific determinants, most biomarkers are best interpreted as context-dependent indicators that integrate diet, absorption, storage, and the acute-phase response.

However, the current biomarker literature has limited specificity and weak causal inference. Many reported associations are cross-sectional, attenuate after adjustment for BMI/adiposity, metabolic syndrome, physical activity, and medication use, and are further complicated by assay variability and the imperfect correspondence between circulating measures and intra-articular concentrations. Sensitivity/specificity thresholds and externally validated cutoffs are rarely established, and direct comparisons showing superiority over established imaging markers are uncommon.

Accordingly, near-term clinical translation should be cautious: nutritional biomarkers are most plausibly used as adjuncts within multimodal panels alongside clinical risk factors and imaging, to support phenotype stratification, identify modifiable systemic contributors, and monitor response in biomarker-enriched interventions, rather than as stand-alone predictors of OA onset or progression.

Future priorities include phenotype- and joint site-stratified longitudinal cohorts with repeated sampling, harmonized assays and pre-specified thresholds, and prediction models reported and externally validated with discrimination, calibration, and decision-curve utility. Integrating synovial-fluid or tissue measurements where feasible, and leveraging causal frameworks (e.g., randomized trials and genetic analyses), will be essential to determine when biomarker changes reflect mechanisms rather than non-specific correlates of comorbidity.

Overall, evidence appears comparatively more consistent for vitamin D status, omega-3-related lipid signatures, and metabolic/AGE-linked measures, whereas several micronutrient and metabolite-derived markers remain context-dependent and require phenotype-specific validation. Translational prioritization should emphasize prospective replication, assay harmonization with predefined cutoffs, and demonstration of added predictive value and clinical utility when integrated with established clinical and imaging variables.
